# Genome-wide methylation in alcohol use disorder subjects: implications for an epigenetic regulation of the cortico-limbic glucocorticoid receptors (NR3C1)

**DOI:** 10.1038/s41380-019-0449-6

**Published:** 2019-06-25

**Authors:** Eleonora Gatta, Dennis R. Grayson, James Auta, Vikram Saudagar, Erbo Dong, Ying Chen, Harish R. Krishnan, Jenny Drnevich, Subhash C. Pandey, Alessandro Guidotti

**Affiliations:** 1 Center for Alcohol Research in Epigenetics, Psychiatric Institute, Department of Psychiatry, College of Medicine, University of Illinois at Chicago, Chicago, IL USA; 2 High-Performance Biological Computing, Roy J. Carver Biotechnology Center, University of Illinois-Urbana Champaign, Urbana, IL USA; 3 Jesse Brown VA Medical Center, Chicago, IL 60612 USA

**Keywords:** Molecular biology, Neuroscience, Molecular biology, Neuroscience

## Abstract

Environmental factors, including substance abuse and stress, cause long-lasting changes in the regulation of gene expression in the brain via epigenetic mechanisms, such as DNA methylation. We examined genome-wide DNA methylation patterns in the prefrontal cortex (PFC, BA10) of 25 pairs of control and individuals with alcohol use disorder (AUD), using the Infinium^®^ MethylationEPIC BeadChip. We identified 5254 differentially methylated CpGs (*p*_nominal_ < 0.005). Bioinformatic analyses highlighted biological processes containing genes related to stress adaptation, including the glucocorticoid receptor (encoded by *NR3C1*). Considering that alcohol is a stressor, we focused our attention on differentially methylated regions of the *NR3C1* gene and validated the differential methylation of several genes in the NR3C1 network. Chronic alcohol drinking results in a significant increased methylation of the *NR3C1* exon variant 1_H_, with a particular increase in the levels of 5-hydroxymethylcytosine over 5-methylcytosine. These changes in DNA methylation were associated with reduced NR3C1 mRNA and protein expression levels in PFC, as well as other cortico-limbic regions of AUD subjects when compared with controls. Furthermore, we show that the expression of several stress-responsive genes (e.g., *CRF*, *POMC*, and *FKBP5*) is altered in the PFC of AUD subjects. These stress-response genes were also changed in the hippocampus, a region that is highly susceptible to stress. These data suggest that alcohol-dependent aberrant DNA methylation of *NR3C1* and consequent changes in other stress-related genes might be fundamental in the pathophysiology of AUD and lay the groundwork for treatments targeting the epigenetic mechanisms regulating *NR3C1* in AUD.

## Introduction

Alcohol use disorder (AUD) is a complex and chronic psychiatric disorder characterized by compulsive alcohol seeking, loss of control over consumption, and a negative emotional state at withdrawal [1, 2]. Individual vulnerability to develop psychiatric disorders depends on an intricate interplay between the genetic background and the impact of the environment. Environmental factors, including substance abuse and stress, cause long-lasting changes in the regulation of gene expression in the brain via epigenetic mechanisms, such as changes in DNA methylation, modifications of histone tails, as well as alterations in the levels of noncoding RNAs [3–9]. Similar to stress, alcohol exposure stimulates the synthesis and release of glucocorticoids—the primary mediators of the stress-response downstream of the hypothalamic–pituitary–adrenal (HPA) axis [10–13]. Glucocorticoids exert their action by binding to specific receptors, i.e., glucocorticoid receptors (encoded by nuclear receptor subfamily 3 group C member 1; *NR3C1*) and mineralocorticoid receptors (encoded by *NR3C2*). These receptors, translocating to the nucleus, act as transcription factors and regulate the expression of glucocorticoid-responsive genes including themselves, corticotropin-releasing factor (*CRF*) [14], and proopiomelanocortin (*POMC*) [15] in a cell-type-specific manner. Importantly, seminal studies demonstrate that perinatal stress induces epigenetic modifications (i.e., increased DNA methylation) of the *NR3C1* gene, resulting in a psychopathological phenotype [16]. It has been suggested that the effects of stress on *NR3C1* promoter methylation serve as an intermediate process by which environmental stimuli result in stable modifications of the phenotype and shape individual responses to stress [3, 17, 18]. The human *NR3C1* gene comprises of eight translated exons (2–9) and nine untranslated alternative first exons (1_A–J_), seven of which are embedded within a CpG island known to be susceptible to epigenetic regulation via DNA methylation [19]. Epigenetic changes of *NR3C1* alternative first exons have been associated with psychopathological conditions in adult human subjects [16, 20].

In animal models of alcohol dependence, reduced *Nr3c1* expression has been observed in stress/reward-sensitive brain regions, such as the prefrontal cortex (PFC) [21], the nucleus accumbens [21], and the paraventricular nucleus of the hypothalamus [22]. Chronic alcohol consumption alters the expression levels of *NR3C1* and stress-response genes in the hippocampus of AUD subjects [23]. Accordingly, chronic treatment with mifepristone, an NR3C1 antagonist, blocks escalated alcohol drinking [21] and reduces the severity of ethanol withdrawal [24] in animal models of dependence. Reduction in alcohol craving and lower alcohol consumption have also been observed in AUD subjects treated with mifepristone [25]. These studies emphasize the importance of NR3C1 signaling in alcohol-drinking behaviors and addictive processes [11, 21, 25].

A reduction in DNA methyltransferase 1 (*DNMT1*) expression and reduced methylation of the long terminal repeat (LTR) retrotransposon has been reported in the brain of AUD subjects [26]. Using a genome-wide promoter methylation microarray, Manzardo and colleagues reported differential methylation hypothetically targeting 3806 genes in the frontal cortex (BA9) of AUD subjects [27]. Another study demonstrated altered DNA methylation levels by using the Illumina HumanMethylation450 BeadChip assay in AUD subjects and found 1812 differentially methylated CpGs (including CpGs located within or near genes), ~66% of which were hypermethylated [28]. Altogether, these studies demonstrate an association between differentially methylated CpG sites and AUD in the PFC [26–28]. However, they do not test whether the hypermethylated genes contain equal or different amounts of 5-methylcytosine (5mC) vs. 5-hydroxymethylcytosine (5hmC) and whether these changes could account for a dysregulation of the HPA axis in AUD. Here, we extended these epigenetic programming findings from behavioral responses to stress to alcohol-induced stress responses, using a genome-wide DNA methylation studying ~850,000 loci and specifically addressed the following questions: is AUD associated with (i) changes in promoter methylation of genes involved in stress responses; (ii) an imbalance of 5mC and 5hmC at the *NR3C1* gene; (iii) alterations of transmethylation reactions in cortico-limbic regions. We report alcohol-induced genome-wide DNA methylation patterns in the PFC (BA10) of 23 pairs of controls and AUD subjects, using the Infinium^®^ MethylationEPIC BeadChip, and validated the results using methyl–DNA–immunoprecipitation and chromatin immunoprecipitation assays. Our data highlight enriched biological processes harboring differentially methylated genes involved in the stress response, including *NR3C1*, that may play a crucial role in the pathophysiology of AUD.

## Subjects and methods

### Subjects

Frozen postmortem brain tissue was obtained from the New South Wales Brain Tissue Resource Centre (NSW BTRC, University of Sydney, Australia) as part of a cohort including 25 control and 25 alcohol use disorder (AUD) subjects described in detail in a previous study [29]. Individuals were diagnosed according to the DSM-IV criteria for AUD [30].

### Bisulfite conversion and genome-wide DNA methylation microarray analysis

DNA was extracted from BA10 samples using QIAamp^**®**^ DNA Mini Kit (Qiagen, Valencia, CA) and bisulfite converted (EZ DNA methylation kit, Zymo Research, Irvine, CA). Genome-wide DNA methylation was assessed by an investigator blind to group allocation using an Infinium^®^ MethylationEPIC BeadChip microarray (Illumina, San Diego, CA), detecting methylation status at over 850,000 loci in the human genome. Statistical analysis of the microarray data was done in R (v 3.4.1) [31]. In brief, we used the minfi package [32] (v 1.22.1) to read in the raw signal intensity data and calculate detection *p*-values. Individual CpG sites that were (i) not detected (*p*-value >0.01) in any sample (6565 CpGs); (ii) located on the X or Y chromosome since our samples contain both males and females (18,977 CpGs); (iii) CpGs with known SNPs at the CpG or the single-base extension site, either of which could alter the perceived methylation signal (28,531 CpGs) [33]; (iv) cross-reactive probes that are known to measure more than one site in the genome (24,739 CpGs) were filtered out. In total, 787,426 CpGs (90.9%) passed all criteria and were retained for further analyses.

Methylated (M) and unmethylated (U) fluorescence values were normalized using quantile normalization [34] and combined into traditional beta-values (proportion of methylation = M/(M + U)) for visualization, while M-values (log ratio of M to U = log_2_(M/U)) were used for analyses [35]. Differential methylation per CpG was tested with the limma package [36] (v 3.32.5) using a model containing subject type (AUD vs. control), slide (eight arrays per slide), and six estimated surrogate variables [37, 38] controlling for correlation structures detected among the samples that were not attributable to subject type or slide. The data discussed in this publication have been deposited in NCBI’s Gene Expression Omnibus [39] and are accessible through GEO Series accession number GSE128401.

### Gene Ontology and Network analysis

Functional analysis was assessed for loci with a *p*_nominal_ ≤ 0.001, which was equivalent to an FDR < 0.703; while this FDR threshold seems extremely high, there were 787,426 CpGs tested, so getting a reasonable FDR threshold is difficult when there are no widespread methylation differences. CpGs with the lowest *p*_nominal_ values have the most evidence for differential methylation and our threshold of *p*_nominal_ ≤ 0.0011 selected only 0.15% (1218 loci) of CpGs for downstream data mining. Out of 1218 loci, 915 were associated with an official gene symbol. Gene ontology classification was assessed using the Panther Classification System. Enriched regulatory pathways were assessed by Kyoto Encyclopedia of Gene and Genomes (KEGG) using Enrichr [40]. Canonical pathways and gene networks were analyzed by QIAGEN’s Ingenuity^®^ Pathway Analysis (IPA^®^, Qiagen, Valencia, CA). Network analysis was used to evaluate highly connected molecules from our list and the QIAGEN knowledge base, with a size constraint of 35 focus molecules per network. Both direct and indirect relationships were considered.

### Methyl–DNA–immunoprecipitation assay

To validate the loci detected in the microarray, we performed methyl–DNA–immunoprecipitation (MeDIP) and hydroxymethyl–DNA–immunoprecipitation (hMeDIP) assays in BA10 using the MagMeDIP kit (Diagenode, Denville, NJ) as previously described [29, 41]. Primers were designed in order to amplify a region including the loci detected by the microarray (*NR3C1*; *SP100; POLR1B; TMOD1; SEMA5B; SAP30BP; LRPPRC*), as well as promoter regions of the *NR3C1* gene (Table S1) [18, 42, 43]. A schematic representation of CpG-rich regions within the *NR3C1* gene is presented in Fig. S1.

### Chromatin immunoprecipitation (ChIP) assay

Occupancy of epigenetic factors modulating DNA methylation was assessed by ChIP. BA10 samples were homogenized, cross-linked with 1% formaldehyde, and quenched with 1 M glycine. Chromatin was sheared using a Covaris sonicator (Covaris, Woburn, MA) to yield ~250-bp DNA fragments. Samples were incubated with an anti-MECP2 antibody (Diagenode, Denville, NJ; #C15410052) or an anti-DNMT1 antibody (Novus Biologicals, Centennial, CO; #NB100–56519). Antibody–chromatin complexes were precipitated using protein A-coupled-Dynabeads^TM^ (Invitrogen, Carlsbad, CA). Immunoprecipitated DNA was concentrated with Chelex^®^ 100 Resin (BioRad, DesPlaines, IL) and proteinase K. Primers are shown in Table S1.

### Reverse transcriptase—quantitative polymerase chain reaction (qRT-PCR)

mRNA levels were measured in BA10, hippocampus, amygdala, and striatum by qRT-PCR following total RNA extraction as previously described [29]. RIN values (controls: 5.93±0.23, AUD: 5.96±0.20) were measured with the Agilent 2100 Bioanalyzer (Agilent Technologies, Santa Clara, CA). Samples with a RIN <3 were excluded from the analyses [29, 41]. Primer sequences used for mRNA expression studies are listed in Table S1. Three reference genes (i.e., beta-2-microglobulin [*B2M*], glyceraldehyde-3-phosphate dehydrogenase [*GAPDH*], and β-actin [*ACTB*]) were chosen for normalization of mRNA levels [29].

### Western blot

Cytosolic and nuclear protein extraction was performed in BA10 as previously described [44]. Validation of western blot with enrichment in nuclear proteins in the soluble nuclear fraction N is shown in Fig. S2. Twenty micrograms of each sample was separated by electrophoresis on Novex 4–12% Tris-Glycine gels (Invitrogen, Carlsbad, CA) and then transferred to a PVDF membrane (Millipore, Billerica, MA). Membranes were incubated with the following primary antibodies: anti-NR3C1 protein (1:1000, Abcam, Cambridge, MA; #ab3671) and anti-GAPDH (1:10,000 for cytosolic protein fraction C, 1:5000 for nuclear fraction N, Millipore, Billerica, MA; #MAB374). HRP-conjugated secondary anti-mouse or anti-rabbit antibodies (1:10,000, GE Healthcare, Arlington Heights, IL) were used and membranes were developed with Immobilon Western Chemiluminescent HRP Substrate (Millipore, Billerica, MA). Densitometric analysis was performed with ImageJ software. Staining of the anti-NR3C1 protein antibody was abolished by blocking with a synthetic peptide (GenScript, Piscataway, NJ) corresponding to human glucocorticoid receptor aa150–175 (Fig. S2).

### Statistical analysis

For all experiments other than the microarray, statistical differences were assessed with two-tailed Student’s *t*-tests and comparisons were considered statistically significant at *p* < 0.05. To control for false discovery rate (FDR), we used the Benjamini–Hochberg [45] approach with FDR, using a level of *Q* *=* 0.1 [29]. ANCOVA was performed for adjusting covariants on the results. Correlation analyses were performed using two-tailed Pearson’s correlation analysis. All statistical tests were run using PASW v.18 software (SPSS).

## Results

### Genome-wide DNA methylation in the PFC of AUD subjects

#### Differentially methylated CpGs

**Fig. 1:**
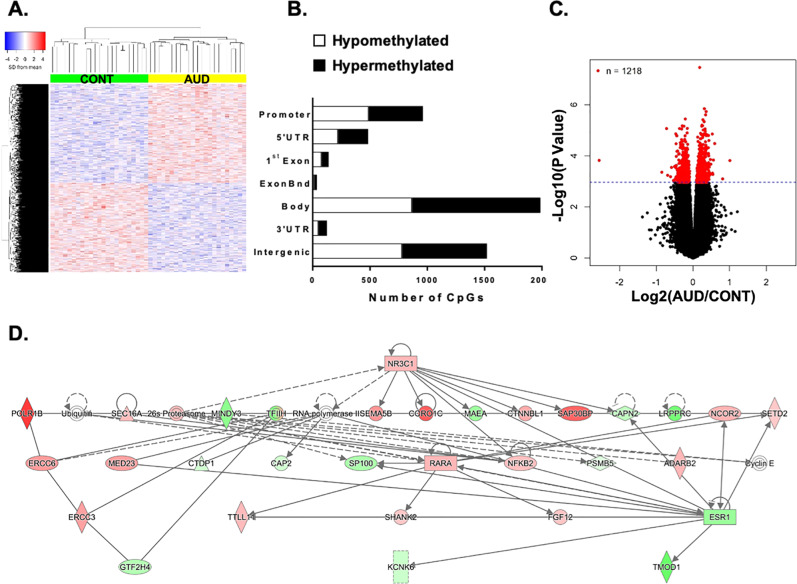
Differentially methylated CpGs in the prefrontal cortex (BA10) of alcohol use disorder (AUD) subjects. **a** Hierarchical clustering of 23 control (green) and 23 AUD (yellow) subjects using a colored heatmap based on methylation levels (blue to red: low-to-high methylation levels) of 5254 CpGs. **b** Location of CpG sites relative to promoters, 5′UTRs, exons, gene bodies, and intergenic regions. Hypomethylated CpGs are shown in white, hypermethylated in black. **c** Volcano plot of effect size [log_2_(fold change)] vs. log_10_(*p*-value) of 787,426 CpGs. Red dots represent 1218 CpGs with *p*_nominal_ ≤ 0.001. **d** IPA^®^ hierarchical interaction network. The lines between genes represent known interactions (solid—direct; dashed—indirect). Genes are referred to as nodes and the intensity of the node color indicates the degree of hyper- (red) or hypo- (green) methylation of a given gene

The genome-wide analyses performed in BA10 tissue of 23 controls and 23 AUD subjects (two pairs of samples were excluded because of low DNA content) showed 5254 differentially methylated CpGs, with 2496 hypomethylated and 2758 hypermethylated loci (*p*_nominal_ < 0.005, Fig. 1a). Out of 5254 individual CpGs, 3734 were located within or near genes: 962 in promoter regions (620 within 1500 bp from the TSS and 342 within 200 bp from the TSS), 484 in 5′UTRs, 139 in first exons, 37 in exon boundaries, 1987 in gene bodies, and 125 in 3′UTRs, while 1520 were located in intergenic regions (Fig. 1b). The distribution of effect size (log_2_(fold change) vs. log_10_(*p*-value)) is represented in the volcano plot (Fig. 1c).

#### Functional annotation

We selected loci with significant differential DNA methylation between controls and AUD that exhibited a *p*_nominal_ < 0.0011 (i.e., 1218 loci associated with 915 gene symbols highlighted in red in Fig. 1c). Panther GO showed several processes of relevance for both alcohol use disorders and stress (Fig. S3, Table S2). Of note, we observed that 40% of genes enriched in “response to stimulus” were involved in “response to stress” (Fig. S3B.5). Enrichment was also observed in “metabolic process” (Fig. S3B.2) and “synapse” (Fig. S3C.7, Table S2) genes. The top 10 categories enriched in KEGG pathways were significant for AUD and stress and included calcium signaling, oxytocin signaling, and the inflammatory pathway (Table S3). IPA^®^ canonical pathway analysis also indicated bias toward addiction and stress, e.g., “CREB signaling in neurons”, “corticotropin releasing hormone signaling” (Fig. S4). The network analysis revealed a top network involved in drug metabolism. The hierarchical clustering of this network indicates a central regulatory role for *NR3C1* (Fig. 1D, Table S4, ID#2). Methylation levels of these genes are presented in Table S5.

#### Validation of methylomic data

**Fig. 2:**
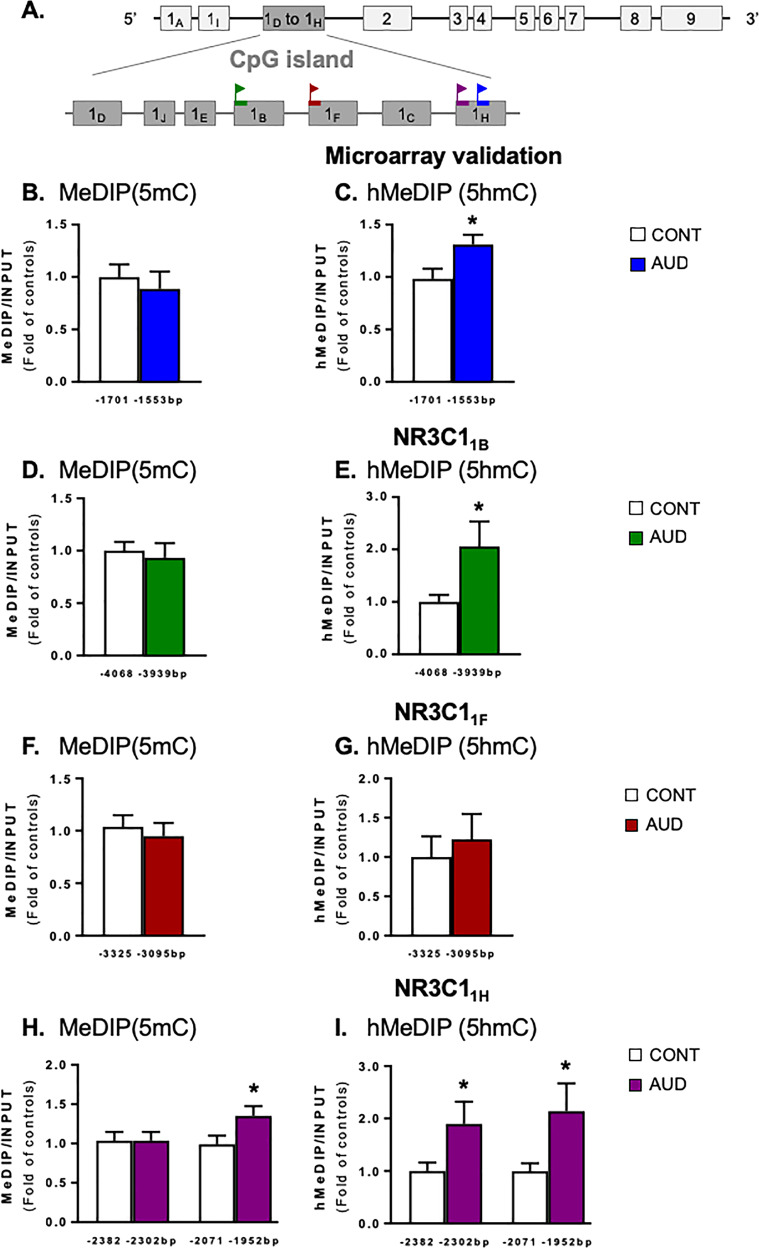
Methylation status of *NR3C1* exon 1 in the prefrontal cortex (BA10) of alcohol use disorder (AUD) subjects. **a** Human *NR3C1* gene structure. Each rectangle is an exon; the CpG island is illustrated as dark gray rectangles. Flag signs indicate the regions of the gene amplified after (hydroxy)methylated DNA immunoprecipitation [(h)MeDIP] assay or chromatin immunoprecipitation (ChIP) in the untranslated alternative first exons. *NR3C1* microarray locus was validated by measuring **b** methylation and **c** hydroxymethylation (*t*_1,44 _= 2.46, *p* = 0.018) levels of a region including the CpG detected in the microarray. *NR3C1*_*B*_ **d** methylation and **e** hydroxymethylation (*t*_1,44 _= 2.16, *p* = 0.036) levels. *NR3C1*_*F*_ **f** methylation and **g** hydroxymethylation levels. *NR3C1*_*H*_ **h** methylation (from –2071 to –1952 bp, *t*_1,44 _= 2.16, *p* = 0.036) and **i** hydroxymethylation levels (from –2382 to –2302 bp: *t*_1,44 _= 2.01, *p* = 0.050; from –2071 to –1952 bp: *t*_1,44 _= 2.02, *p* = 0.048). Values are mean±SEM of 23 samples per group for (h)MeDIP assay, 25 controls, and 24 AUD subjects for ChIP. *p < 0.05 Student’s *t*-test vs. controls

We subsequently validated the differentially methylated probes present on the EPIC array corresponding to *NR3C1* (–1669 bp with respect to the translation initiation site). We used MeDIP and hMeDIP to amplify a region inclusive of this locus (from –1701  to –1553 bp) within exon 1 _H_ of the *NR3C1* gene, a region rich in CpG sites (see Fig. 2a for gene structure). In this region of the *NR3C1* gene, we observed higher levels of 5hmC but not 5mC (Fig. 2b, c, Table S6). A significant negative correlation was detected between the levels of 5hmC and age (*r* = –0.452, *p* = 0.001), but when age was adjusted, the difference between groups remained significant (*F*_1,47_ = 7.39, *p* = 0.009). A significant negative correlation also existed between the levels of 5hmC and the number of drinks per week (*r* = –0.296, *p* = 0.046). No correlation with other demographic variables was observed. Validation of DNA differential methylation levels for several selected genes (i.e., *SP100; POLR1B; TMOD1; SEMA5B; SAP30BP;* and *LRPPRC*) included in the IPA^®^ network analysis was also assessed (Table S6). We were able to associate changes observed in methylation levels with increases and decreases in 5mC or 5hmC enrichment in a region of the genome inclusive of the loci detected by the microarray.

#### Promoter methylation

Next, we further investigated the methylation status of the *NR3C1* exon 1 in control and AUD subjects. While no changes in 5mC levels were found in the promoter region of the untranslated exons 1_B_ and 1_F_ (Fig. 2d, f, respectively), there were increased 5mC levels at exon 1_H_ (∼35%, Fig. 2h). Higher levels of 5hmC were found for exon 1_B_ (Fig. 2e) and 1_H_ (Fig. 2i) in AUD subjects. Interestingly, a significant correlation was found between 5hmC levels of exon 1_B_ and 1_H_ (both fragments), and ethanol daily use (g) (*r* = 0.397, *p* = 0.006; *r* = 0.403, *p* = 0.006; *r* = 0.431, *p* = 0.003, respectively), as well as between 5hmC levels of exon 1_B_ and 1_H_ (both fragments), and the number of standard drinks per week (*r* = 0.477, *p* = 0.001; *r* = 0.413, *p* = 0.004; *r* = 0.518, *p* = 0.0001, respectively). No significant correlation was observed between 5hmC levels and PMI or pH. No apparent influence of sex was found, but the significance of this observation remains inconclusive due to the small number of females in our cohort (*n* = 4).

### Transcriptional changes of NR3C1 in AUD subjects

**Fig. 3:**
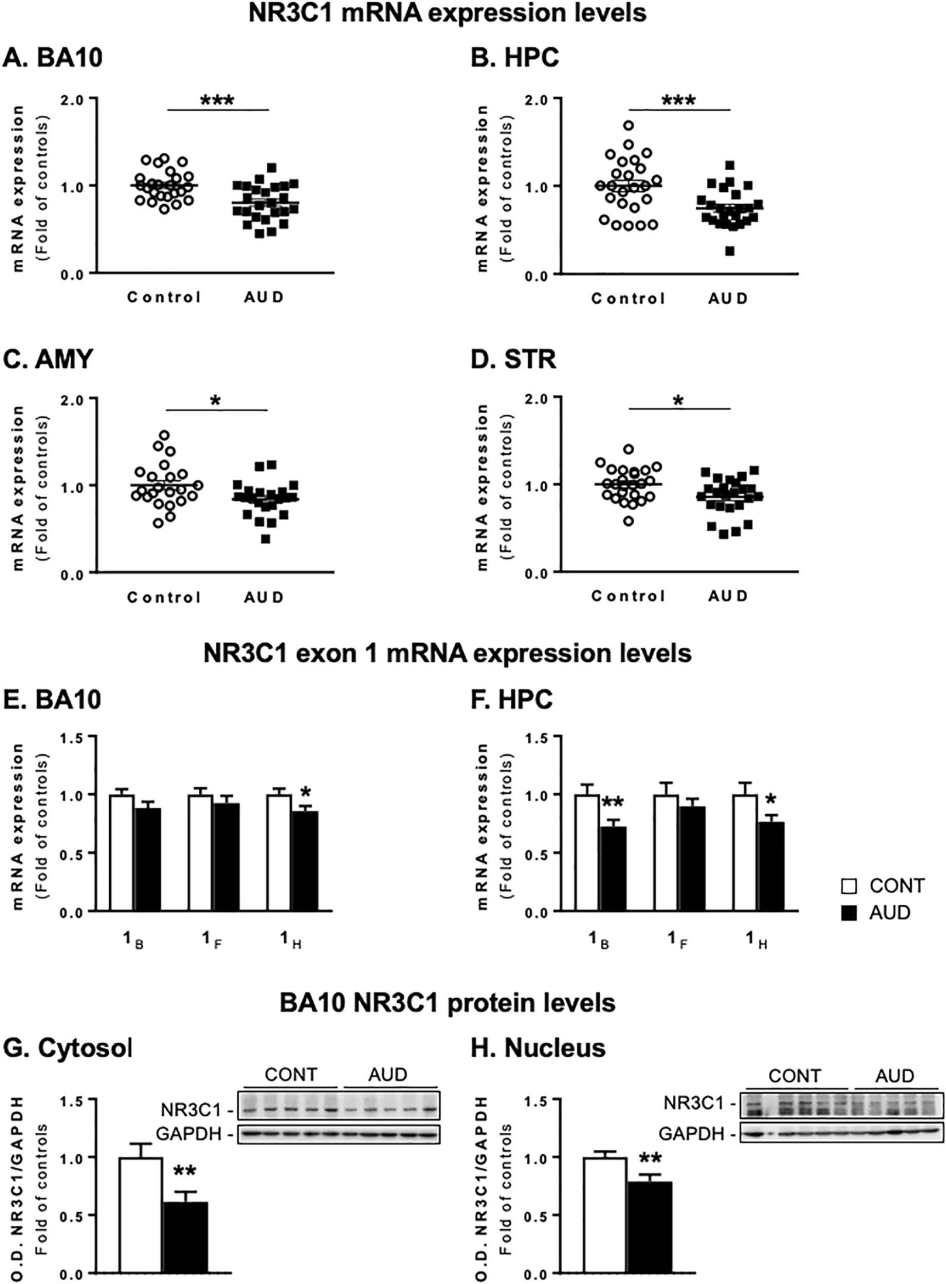
NR3C1 expression levels in alcohol use disorder (AUD) subjects. *NR3C1* mRNA levels are expressed as fold change of controls in **a** prefrontal cortex (BA10, *t*_1,46 _= 3.75, *p* = 0.0005), **b** hippocampus (HPC, *t*_1,46 _= 3.28, *p* = 0.002), **c** amygdala (AMY, *t*_1,46 _= 2.39, *p* = 0.022), and **d** striatum (STR, *t*_1,46 _= 2.55, *p* = 0.014). *NR3C1* exon *1*_*B, F*, *and H*_ are shown in **e** the BA10 (*NR3C1*_*1H*_: *t*_1,46 _= 2.18, *p* = 0.034) and **f** the HPC (*NR3C1*_*1B*_: *t*_1,46 _= 2.72, *p* = 0.009; *NR3C1*_*1H*_: *t*_1,46 _= 2.08, *p* = 0.042). Protein levels of NR3C1 measured as a ratio of NR3C1/GAPDH optical density (O.D.) values in **g** cytosolic (*t*_1,46 _= 2.68, *p* = 0.010) and **h** nuclear (*t*_1,46 _= 2.79, *p* = 0.007) fractions are expressed as fold of controls. Representative western blots are shown on the right side. Values are mean ± SEM of 24 samples per group for mRNA levels and 25 samples per group for the protein analysis. *p < 0.05, **p < 0.01, Student’s *t*-test vs. controls

To investigate whether the observed methylation changes were also associated with mRNA changes in AUD subjects, we assessed the expression of *NR3C1* as measured by the total mRNA levels and transcript containing different untranslated alternative first exons (1_B_, 1_F_, and 1_H_). Our data show that the total *NR3C1* mRNA levels were highly reduced in BA10 (Fig. 3a), hippocampus (Fig. 3b), amygdala (Fig. 3c), and striatum (Fig. 3d) of AUD subjects. These changes were associated with reduced expression of *NR3C1*_*1H*_ in BA10 (Fig. 3e) and hippocampus of AUD subjects, where *NR3C1*_*1B*_ was also reduced (Fig. 3f). In BA10, protein levels of NR3C1 were reduced in AUD subjects in both cytosolic (Fig. 3g) and nuclear (Fig. 3h) fractions.

### Altered regulation of DNA methylation/demethylation processes in AUD subjects

**Fig. 4:**
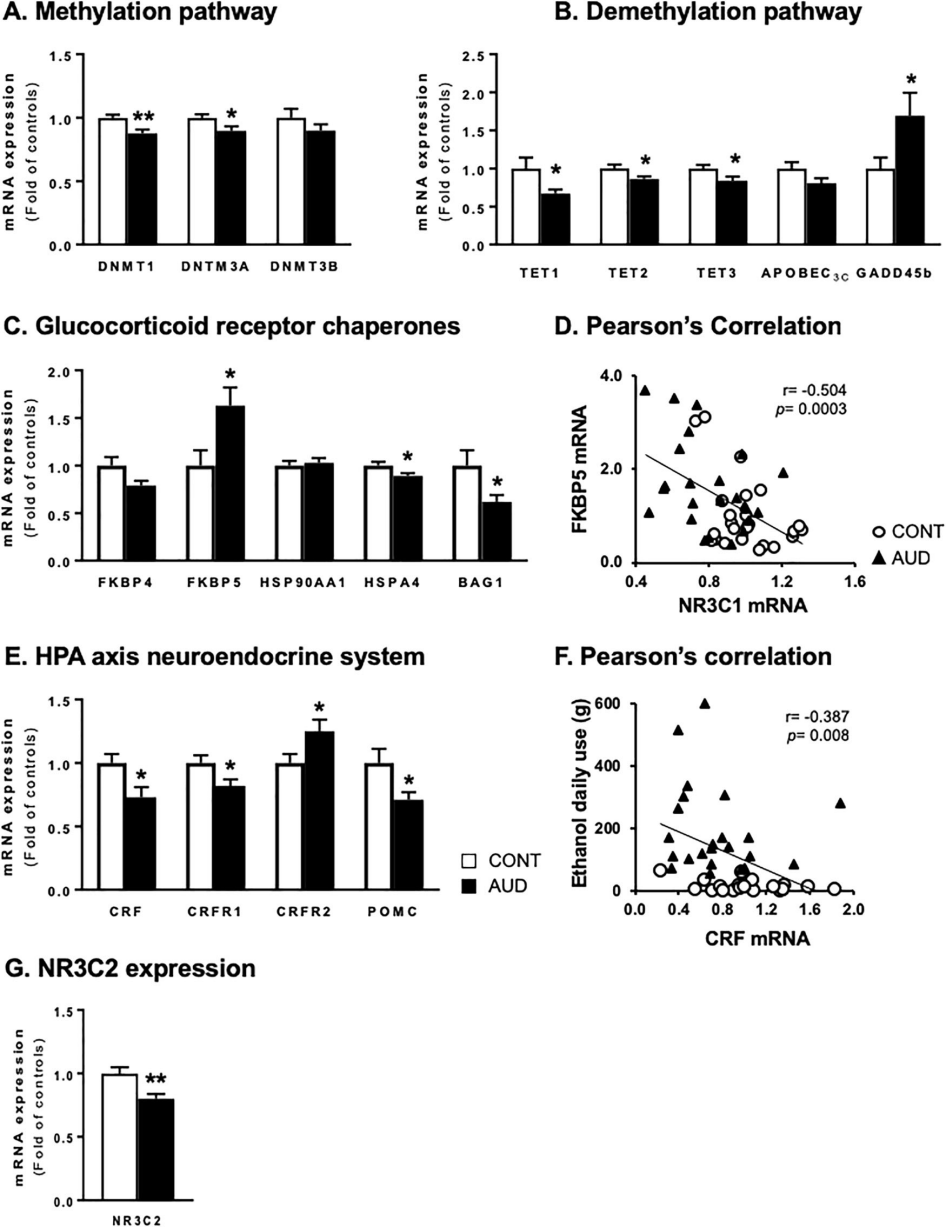
Transcriptomic changes in the stress system and DNA transmethylation reactions in the prefrontal cortex (BA10) of alcohol use disorder (AUD) subjects. mRNA levels of **a** enzymes of the DNA methylation pathway, i.e., DNA methyltransferases (*DNMT*) *1* (*t*_1,46 _= 3.16, *p* = 0.003), –*3A* (*t*_1,46 _= 2.28, *p* = 0.027), and –*3B*. These data remained significant after controlling for FDR (adjusted *p*-value = 0.003, 0.015, respectively). **b** Enzymes involved in DNA demethylation pathway, such as Ten-Eleven Translocase (*TET*) *1* (*t*_1,46 _= 2.17, *p* = 0.035), *–2* (*t*_1,46 _= 2.25, *p* = 0.029), and –*3* (*t*_1,46 _= 2.20, *p* = 0.034), as well as apolipoprotein B mRNA editing enzyme catalytic subunit 3C (*APOBEC 3C*) and growth arrest and DNA damage-inducible beta (*GADD45B*, *t*_1,46 _= 2.07, *p* = 0.044). These data remained significant after controlling for FDR (adjusted *p*-value = 0.061). **c** Glucocorticoid receptor cytosolic chaperones: FK506 binding protein 4 (*FKBP4*) and 51 (*FKBP5*, *t*_1,46 _= 2.49, *p* = 0.016), heat-shock protein 90 alpha family class A member 1 (*HSP90AA1*), heat-shock protein family A (Hsp70) member 4 (*HSPA4*, *t*_1,46 _= 2.11, *p* = 0.039), BCL2-associated athanogene 1 (*BAG1*, *t*_1,46 _= 2.16, *p* = 0.036). These data remained significant after controlling for FDR (adjusted *p*-value = 0.014). **d** Pearson’s correlation analysis of *FKBP5* and *NR3C1* mRNA levels. **e** Hypothalamic–pituitary–adrenal (HPA) axis neuroendocrine system: corticotropin-releasing factor (*CRF*, *t*_1,46 _= 2.52, *p* = 0.015), corticotropin-releasing factor receptor 1 (*CRFR1*, *t*_1,46 _= 2.19, *p* = 0.034), corticotropin-releasing factor receptor 2 (*CRFR2*, *t*_1,46 _= 2.19, *p* = 0.033), and proopiomelanocortin (*POMC*, *t*_1,46 _= 2.31, *p* = 0.026). These data remained significant after controlling for FDR (adjusted *p*-value = 0.037). **f** Pearson’s correlation analysis of *CRF* mRNA levels and ethanol daily use (g). **g** Mineralocorticoid receptor (*NR3C2*) mRNA expression levels (*t*_1,46_ = 3.19, *p* = 0.002). Values are mean ± SEM of 24 samples per group. *p < 0.05, **p < 0.01, Student’s *t*-test vs. controls

To characterize the factors controlling the enrichment of 5mC and 5hmC, we first measured the steady-state levels of the methyl donor S-adenosyl methionine (SAM, control: 1.34±0.27; AUD: 0.99±0.19 ng) and S-adenosyl-L-homocysteine (SAH, control: 1.88±0.22; AUD: 1.68±0.19 ng). These levels were similar in control and AUD subjects. Within the one-carbon metabolism enzymes, only the adenosyl-homocysteinase was increased in the BA10, while the methylenetetrahydrofolate reductase was increased in the hippocampus of AUD subjects (Fig. S5). In addition, the mRNA levels of DNA-methylating enzymes *DNMT1* and *DNMT3A* were slightly but significantly reduced in BA10 of AUD subjects (Fig. 4a). Of note, the binding of DNMT1 to exon 1_H_ of *NR3C1* was reduced by about ∼35% in individuals with AUD (Table S7), despite the increased 5mC and 5hmC levels in the same region. Furthermore, a significant inverse correlation was found between *DNMT1* mRNA in BA10 and drinking-related measures (i.e., ethanol daily use (g): *r* = –0.441, *p* = 0.005; number of drinks per week: *r* = –0.377, *p* = 0.010). No correlation was observed with PMI. In addition, higher methylation levels were also observed in the EPIC array for *DNMT3B* in AUD subjects (fold change = 0.18; *p*_nominal_ = 0.0004; FDR = 0.663).

Importantly, reduced mRNA levels of the hydroxymethylating enzymes, such as *TET1*, *TET2*, and *TET3* were observed in the BA10 of AUD subjects (Fig. 4b). Conversely, levels of growth arrest and DNA damage-inducible beta (*GADD45B*), a protein involved in deamination processes, were increased in BA10 of AUD subjects (Fig. 4b).

Significant correlations were noted between pH and *DNMT3A* (*r* = 0.469, *p* = 0.001), as well as pH and *TET1* mRNA (*r* = –0.332, *p* = 0.021). Hence, changes in *DNMT3A* and *TET1* were tested using ANCOVA. After adjusting for pH effects, the differences in *DNMT3A* and *TET1* mRNA expression observed between groups remained significant (*F*_1,47_ = 5.89, *p* = 0.019; *F*_1,47_ = 5.76, *p* = 0.021, respectively). *GADD45B* correlated with age (*r* = 0.457, *p* = 0.001); however, correcting for age, the ANCOVA analysis showed that the difference in *GADD45B* mRNA between groups remained significant (*F*_1,47_ = 5.65, *p* = 0.022).

In exons with increased 5hmC, we also found reduced levels of MECP2 binding in AUD subjects (∼30–40%, Table S7), suggesting a complex alteration of DNA methylation/demethylation processes in AUD. MECP2 binding at exon 1_F_ was below detection limits in our experimental conditions. No changes in *MECP2* mRNA levels were observed (Fig. S6). A significant correlation was observed between MECP2 binding at exon 1_H_ and pH (*r* = 0.317, *p* = 0.026; *r* = 0.367, *p* = 0.010). Adjusting for this covariant did not affect the difference between groups (*F*_1,49_ = 9.55, *p* = 0.003; *F*_*1*,49_ = 5.62, *p* = 0.022).

### Transcriptional changes of stress-related genes in AUD subjects

To further investigate NR3C1/NR3C2 regulation, the mRNA levels of receptor-associated chaperone proteins were also assessed. In BA10, increased mRNA levels of FK506 binding protein 5 (*FKBP5*) and reduced mRNA levels of heat-shock protein family A (*Hsp70*) member 4 (*HSPA4*) and BCL2-associated athanogene 1 (*BAG1*) were observed in AUD subjects (Fig. 4c). Similar changes were found in the hippocampus (Fig. S7C) and amygdala (*FKBP5* mRNA: 1.69±0.28, *t*_1,46_ = 2.39, *p* = 0.022) of AUD subjects. In BA10 (Fig. 4d), hippocampus (Fig. S7B), and amygdala (*r* = –0.330, *p* = 0.037), *NR3C1* levels were inversely correlated with *FKBP5* mRNA. Moreover, in BA10, the methylation levels of the *FKBP5* promoter were not different (*p* = *0.132*) in AUD subjects (mean methylation levels fold of control 0.82±0.09). No differentially methylated loci within the *FKBP5* gene were observed in our microarray.

To further assess the changes in “corticotropin releasing hormone signaling” identified in our IPA^®^ analysis, we also measured mRNA levels of genes in the hypothalamic–pituitary–adrenal (HPA) axis neuroendocrine system. In BA10, we showed reduced mRNA levels of *CRF*, *CRFR1*, and *POMC*, while *CRFR2* is increased (Fig. 4e). Similar changes were observed in the hippocampus (Fig. S7E). Of note, a significant correlation between *CRF* mRNA levels and ethanol daily use (g) was detected in BA10 (Fig. 4f) and hippocampus (Fig. S7F). In addition, changes in methylation levels were also observed in the EPIC array for the high-affinity mineralocorticoid receptor encoded by *NR3C2* (fold change = 0.16; *p*_nominal_ = 0.002; FDR = 0.729). mRNA levels of this gene were reduced in BA10 (Fig. 4g), but not in the hippocampus of AUD subjects (Fig. S7G).

## Discussion

This study reports a differential pattern of genome-wide DNA methylation in BA10 of AUD subjects, indicating that the majority of the highly significant differentially methylated CpGs (~61% of 1218 CpGs), including the CpG of *NR3C1*_*1H*_, were hypermethylated in AUD subjects. A large proportion (~60%) of differentially methylated genes detected in BA10 of our cohort also showed differential transcriptome expression in a study that Farris and collaborators conducted in BA8 of a NSW BTRC AUD cohort [46]. Bioinformatic analyses of the differentially methylated CpGs of our data set identified categories containing genes related to stress adaptation, inflammatory cascade, metabolic processes, transcription factors, and neurotransmission. Based on these analyses and considering that alcohol is a stressor, which leads to glucocorticoid secretion [11, 12, 47, 48], we focused our attention on differentially methylated regions of the *NR3C1* gene (Fig. 2) known to be regulated by promoter methylation [16]. We observed that chronic exposure of adult subjects to large doses of alcohol results in a significant increased methylation of the *NR3C1*_*1H*_ exon variant with a particular increase in 5hmC over 5mC. The hyper-/hydroxy-/methylation of *NR3C1* induced by chronic alcohol consumption preferentially targets the exon *1*_*H*_, while the *1*_*F*_ and *1*_*C*_ exon variant are reported as differentially methylated in suicidal patients [18, 42] and in women with bulimia nervosa [43], respectively. It should be noted that no measurements of 5hmC were performed in these two cohorts.

Using the precursor platform (Infinium^®^ HumanMethylation450K BeadChip), Wang et al. did not report a change in *NR3C1* methylation in the PFC (BA9) of tissue obtained from NSW BTRC [28]. However, of the 1812 differentially methylated CpGs they reported in males with AUD, only 1708 were present on the EPIC array and did not include neither *NR3C1* nor *NR3C2* after filtering. Despite the high FDR values that could be due to testing almost 800,000 loci and also demographic variables, such as age, sex, and other variables that are unknown to the investigators, results indicating a differential methylation of *NR3C1*_*1H*_ and several other stress-related genes observed in the EPIC array were also validated when measured by methylated DNA immunoprecipitation assay with specific antibodies against 5mC or 5hmC.

**Fig. 5:**
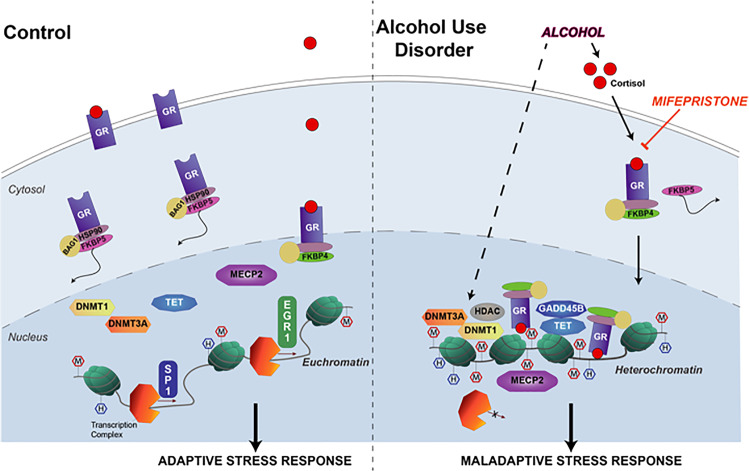
Schematic representation of chronic alcohol-induced stress response via an epigenetic regulation of glucocorticoid receptor expression. An adaptive response to stress is controlled by the glucocorticoid receptor (GR), the low-affinity receptor for cortisol. In unstressed control conditions (left panel) with low levels of cortisol, GRs are not translocated to the nucleus and their expression is regulated by transcription factors, such as specificity protein 1 (SP1) and nerve growth factor-induced protein A (EGR1). Alcohol consumption, similar to stress, leads to enhanced levels of cortisol, which enters the neurons and binds to the GR (right panel). The activation and nuclear translocation of the GR is crucial for the control of the stress response and is regulated by molecular chaperones (i.e., heat-shock protein 90 alpha family class A member 1, HSP90; BCL2- associated athanogene 1, BAG1; FK506 binding protein 4, FKBP4; and FK506 binding protein 51, FKBP5). Once bound to the chromatin, GR recruits chromatin-remodeling factors, including DNA methyltransferases (DNMT1 and –3A) and demethylating (ten–eleven translocases, TET1–3; growth arrest and DNA damage-inducible beta, GADD45B) enzymes, histone deacetylases (HDAC), and methyl CpG binding protein 2 (MECP2). By remodeling chromatin, excessive alcohol consumption results in the negative epigenetic regulation of the expression of glucocorticoid-sensitive genes, including the GR gene (nuclear receptor subfamily 3 group C member 1, *NR3C1*) itself, that may play an important role in the overall dysregulation of the stress response and pathophsiology of alcohol use disorders. This results in an altered promoter methylation (M: CpG methylation; H: CpG hydroxymethylation), leading to a maladaptive stress response in alcohol use disorder subjects

The heightened 5hmC/5mC ratio at the *NR3C1*_*1H*_ promoter we noted in PFC of AUD subjects is likely due to the accumulation of 5hmC levels resulting from the reduced TET1 expression, which prevents the further processing of 5hmC into 5-carboxylcytosine (5caC) and the successive progression of 5caC to 5 C by the base-excision repair pathway. Hence, our findings suggest that chronic alcohol may act on the *NR3C1* gene by altering the levels of methylation (DNMTs) and demethylation (TETs) enzymes (Fig. 4), resulting in an imbalance of methylation turnover. We are aware that the experiments carried out in postmortem brain of individuals with AUD only provide a snapshot of the dynamic equilibrium regulating 5mC/5hmC at specific locations in the genome. The reduced DNMT1 binding to *NR3C1*_*1H*_ in the presence of increased 5mC and 5hmC levels suggests the existence of a molecular mechanism mediated by TET1 which maintains hydroxymethylation levels of *NR3C1* to an abnormal steady state. Thus, chronic alcohol consumption may influence gene transcription via at least two mechanisms: (i) DNA methylation that inhibits the binding of transcription factors to promoter regions and thus directly interferes with gene transcription, or (ii) DNA methylation attracts CpG binding proteins to promoter regions and recruits a variety of histone deacetylases or methylases and chromatin-remodeling epigenetic factors indirectly facilitating chromatin compaction and transcriptional repression (Fig. 5). Since 5hmC is observed at higher levels in the brain than in peripheral tissues, one can infer that 5hmC is not merely an intermediate product of DNA methylation, but might also regulate neuronal development and synaptic plasticity in subjects with chronic alcohol consumption [49]. Our observation of lower MECP2 binding at promoters with higher 5hmC levels agrees with previous reports showing that 5mC conversion into 5hmC substantially decreases MECP2 affinity for DNA [50]. The classical epigenetic model of MECP2-dependent disorders supports the concept that decreased MECP2 is associated with increased gene expression. However, strong evidence indicates that MECP2 can contribute to both activation and repression of gene transcription [51, 52]. The study of DNA methylation levels in the brain needs to take into consideration that the expression of methylated or unmethylated cytosines is cell specific and that methylation levels detected in the brain are representative of the whole brain cellular populations. Therefore, further studies should assess *NR3C1* promoter methylation pattern in a cell-specific manner.

The functional role of an alternative exon 1 variant methylation in response to different types, duration and frequency of stress, as well as their relation to the risk of psychiatric disorders, suggests the existence of a site-specific methylation process at the level of *NR3C1*. The alternative *NR3C1* first exons have different transcription factor-binding sites, e.g., *1*_*B*_ has consensus sequences for specificity protein 1 (SP1) [53], whereas nerve growth factor-induced protein A (EGR1) binds to *1*_*F*_ [54]. While the expression of EGR1 is not changed (AUD mRNA expression: 0.83±0.09), SP1 is decreased in BA10 of AUD subjects (0.81±0.04, *t*_1,46_ = 2.20, *p* = 0.033). We expect that the intrinsic spatial and temporal expression properties of these two transcription factors may play an important role in *NR3C1* expression.

In response to the alcohol-induced hypermethylation of the *NR3C1* exon 1 variant and consequent reduction of *NR3C1* mRNA and protein, we observed an increase of *FKBP5* mRNA both in the BA10 and the hippocampus of AUD subjects. FKBP5 is a regulator of NR3C1 sensitivity to glucocorticoids, making it an important negative modulator of the HPA axis [55–57]. NR3C1 activation by alcohol results in rapid induction of FKBP5, which binds to NR3C1 and decreases its ability to bind cortisol and subsequently translocate to the nucleus [56] (Fig. 5). Although studies suggest that rapid increases in FKBP5 could result from promoter demethylation [56], more work is needed to clarify patterns of (de)methylation following chronic alcohol exposure.

Here, we have shown that several stress-responsive genes, including *CRF*, *POMC*, *NR3C1*, and *NR3C2*, that are vital for the regulation and function of the HPA axis, are altered in the PFC and hippocampus of AUD subjects. Regulation of this system is complex, but promoter hypo-/hypermethylation might be a major epigenetic mechanism responsible for changes in the stress pathways. Alcohol exposure leads to secretion of cortisol which enters the brain and binds to cytosolic NR3C1 protein. When translocating to the nucleus, NR3C1 acts as a transcription factor and becomes the host for the recruitment of a chromatin-remodeling repressor protein complex (containing both MECP2 and DNMTs [58]), which may induce epigenetic modifications on target genes [57], including *NR3C1* itself (Fig. 5). To better interpret the changes in the brain stress responses in AUD subjects, we need to consider the progression of alcohol dependence over time [21]. The allostatic load induced by excessive and repetitive alcohol consumption results in maladaptive epigenetic glucocorticoid receptor regulation. However, our data examine only one time point and do not preclude the existence of an epigenetic programming that predisposes to AUD. This study suggests that alcohol-dependent epigenetic regulation of *NR3C1* expression and consequent changes in other stress-related genes in stress/reward-responsive brain regions, such as the PFC and the hippocampus, might be involved in the pathogenesis of AUD. These findings lay the groundwork for the development of treatments targeting the aberrant epigenetic regulation of NR3C1 in AUD.

## Supplementary information

Supplementary tables and figures
