# Supplementary material for: Genome-wide methylation in alcohol use disorder subjects: implications for an epigenetic regulation of the cortico-limbic glucocorticoid receptors (NR3C1)
Source: Mol Psychiatry. 2019 Jun 25;26(3):1029–41. doi: 10.1038/s41380-019-0449-6 (PMC6930366; doi:10.1038/s41380-019-0449-6)
Supplement: Supplementary file 1 — Supplementary tables and figures [file 41380_2019_449_MOESM1_ESM.doc]

**Methods**

***High-performance liquid chromatography (HPLC)***

S-adenosyl-methionine (SAM) and S-adenosyl-homocysteine (SAH) levels were determined using a reverse-phase HPLC column, Symmetry C18 4.6 x 250 nm (Waters, Milford, MA). Samples were homogenized in 0.4 M HClO_4_ as previously described.^29^ Five-point calibration curves for SAM and SAH (Sigma-Aldrich, St. Louis, MO) were assessed in the range of 25-800 ng of the respective standards.

**Table S1. Primer Sequences.**

| **Gene symbol** | **Forward** | **Reverse** | |
| --- | --- | --- | --- |
| *mRNA expression* |  |  | |
| ***ACTB*** | 5'-CTCCCTGGAGAAGAGCTAC | 5'-GATCCACACGGAGTACTTG | |
| ***AHCY*** | 5'-GACCGGTATCGGTTGAAGAATGG | 5'-GGTACTTGTCTGGATGGGTCCA | |
| ***APOBEC3C*** | 5'-TGTATCCAGGCACATTCTACTTC | 5'-AACTGAGCGGCGCTTTAT | |
| ***B2M*** | 5'-CTATCCAGCGTACTCCAAA | 5'-GCTCCACTTTTTCAATTCTC | |
| ***BAG1*** | 5'-ATCCTCTCTCGACTTCCCATAC | 5'-CCAAGGAACCCTGGTGTTAAA | |
| ***CRF*** | 5'-GAGAGAGGGAGAGAGCCTATAC | 5'-CACCTGGAAACGGAAACTAAAC | |
| ***CRFR1*** | 5'-CTATGGTGTCCGCTACAATACC | 5'-CCCAGGTAGTTGATGATGACTG | |
| ***CRFR2*** | 5'-TCTCGTGCTCCTGATCAATTTC | 5'-GTACTGGATTGTCTCGGATGTG | |
| ***DNMT1*** | 5'-AGAAGCTGTCCATCTTTGAT | 5'-CATAGATTGGTTTTGCTGAA | |
| ***DNMT3A*** | 5'-TCTTTGATGGAATCGCTAC | 5'-GCGGTAGAACTCAAAGAAGA | |
| ***DNMT3B*** | 5'-GCTCTTACCTTACCATCGAC | 5'-GAGACGAGCTTATTGAAGGT | |
| ***EGR1*** | 5'-TAAAGGACAGGAGGAGATG | 5'-GGAAGTGGGCAGAAAGGATT | |
| ***FKBP4*** | 5'-GTCTGGGTCCTCTTGGTATTTC | 5'-CAGTGCTAGGCTTACTCCATAAA | |
| ***FKBP5*** | 5'-GTCTCCCACGTGTGTATTATGG | 5'-TGGGCACCCTGTAGTTATTTG | |
| ***GADD45b*** | 5'-ATTGCAACATGACGCTGGAA | 5'-CTGTCTGGGTCCACATTC | |
| ***GAPDH*** | 5'-CGAGATCCCTCCAAAATCAA | 5'-TTCACACCCATGACGAACAT | |
| ***HSPA4*** | 5'-GACCTGCCAATCGAGAATCA | 5'-CCGCTCCTTCTCCAGTTTATC | |
| ***HSP90AA1*** | 5'-GGAGATAAACCCTGACCATTCC | 5'-GACAGGAGCGCAGTTTCATA | |
| ***MAT2A*** | 5'-GATGCCAAAGTAGCTTGTGAAACT | 5'-CGTTACAAGTCTTGTAGTCAAAACCT | |
| ***MAT2B*** | 5'-GAGAACAATCTAGGAGCTGCTGT | 5'-CCTTAATTGATGGATCCAGCATTCTC | |
| ***MTHFR*** | 5'-CTTGAAGGAGAAGGTGTCTGCG | 5'-ATCTCCTGTGGCACCTCCA | |
| ***MTR*** | 5'-CGCAACCCGAAGGTCTGAA | 5'-TGGTAAATGACATCAGGCTGAGTTATA | |
| ***NR3C1*** |  |  | |
| Total | 5'-CAGCTCCTCAACAGCAACAACA | 5'-GTGCTGTCCTTCCACTGCTCT | |
| *1_B_* | 5'-CCGGGCCCAAATTGATATTCACT | 5'-GTCTTCGCTGCTTGGAGTCTG | |
| *1_F_* | 5'-GCGGGAAGGAGGTAGCGA | 5'-CCTTCACAGTAGCTCCTCCTCT | |
| *1_H_* | 5'-TGACAGCCCGCAACTTGG | 5'-CAGTAGCTCCTCCTCTTAGGGT | |
| ***NR3C2*** | 5'-CAGCTCTCCACAATCTCACGAG | 5'-CTGGAAGTACCTTTGCCCACT | |
| ***POMC*** | 5'-GTTTCATGACCTCCGAGAAGAG | 5'-TGTAGGCGTTCTTGATGATGG | |
| ***SP1*** | 5'-CTGAAGCTGGGTAGCCTATTG | 5'-CTACTGCTGCGACCTTTCTT | |
| ***TET1*** | 5'-CCCGGGCTCCAAAGTTGTG | 5'-GCAGGAAACAGAGTCATT | |
| ***TET2*** | 5'-TGTGTGGCACTAGATTTCAT | 5'-AGTCTCTGAAGCCTGTTGAT | |
| ***TET3*** | 5'-CAGTGGCTTCTTGGAGTCACCTC | 5'-GGATGGCTTTCCCCTTCTCTCC | |
| *FKBP5 promoter region* | | | |
| **FKBP5-207-68bp** | 5’-AGGTGGAGGAGCAAATGATG | 5’-AGGACCCGCCTTCCATA |  |
| *NR3C1 Methylated DNA and Chromatin Immunoprecipitation* | | | |
| ***1_B_*** -**4068 -3939 bp** | 5'-CTATGAACGTGATAGGGTGAGC | 5'-CGAAACTCCTGACCTCTTCTTC | |
| ***1_F_*** **-3325 -3095 bp** | 5'-TCTTGTGGCCCGCCCGCTGTCA | 5'-CCGCCCCCAACTCCCCAGGAA | |
| ***1_H_*** **-2071 -1952 bp** | 5'-GGGAATCCTGGCCTCTTT | 5'-GCAAATATTCGGGCGAGTAAA | |
| ***1_H_*** **-2382 -2302 bp** | 5'-CTGCCAGAGGTAAGAAGCG | 5'-AGTTGTCTCCGGTCCCA | |
| EPIC array validation | | | |
| ***1_H_*** **-1701 -1553 bp** | 5'-GCTTGCTCTTTAGCGTTTGTT | 5'-GCAATTATCAAGTCTGCAACCC | |
| ***SP100*** | 5’-CTGAATCCTCACCAGCTTCC | 5’-CACAACCAGGGACCAGAAC | |
| ***POLR1B*** | 5’-GGCGGAATATGGGAGAAAGG | 5’-CCTGCGGTTTCGGTTTCT | |
| ***LRPRRC*** | 5’-CCTCCTAAGAAGCTAACCACAC | 5’-GACACCTGTCTCCAATTCCTT | |
| ***TMOD1*** | 5’-GCCACACTCTCAGGCTAAA | 5’-CACTTATGGTCCCATCCCAATA | |
| ***SEMA5B*** | 5’-GGGCTGAATTCTGAATGGC | 5’-CTTCAGGTACGATTGCTTTCTTC | |
| ***SAP30BP*** | 5’-AAAGCCCTCAGCCTGTTC | 5’-CACCAGGCATCTGTCTACATC | |

**Table S2. Differential Methylated CpGs Panther Gene Ontology Classification.**

Most enriched categories are shown for Molecular Function, Biological Process, Cellular Component.

| **Sub-root** | **Gene Ontology Term** | **Percent of Gene Hit Against Total Number of Genes** |
| --- | --- | --- |
| **Molecular Function** | | |
| *binding (GO:0005488)* | protein binding (GO:0005515) | 54.9 |
|  | nucleic acid binding (GO:0003676) | 28.3 |
|  | calcium ion binding (GO:0005509) | 5.6 |
|  | lipid binding (GO:0008289) | 3.9 |
|  | nucleotide binding (GO:0000166) | 3.9 |
|  | chromatin binding (GO:0003682) | 2.6 |
|  | calcium-dependent phospholipid binding (GO:0005544) | 0.9 |
| *catalytic activity (GO:0003824)* | hydrolase activity (GO:0016787) | 41.7 |
|  | transferase activity (GO:0016740) | 27 |
|  | enzyme regulator activity (GO:0030234) | 9.5 |
|  | oxidoreductase activity (GO:0016491) | 7.6 |
|  | ligase activity (GO:0016874) | 6.6 |
|  | lyase activity (GO:0016829) | 3.8 |
|  | isomerase activity (GO:0016853) | 1.9 |
|  | helicase activity (GO:0004386) | 1.4 |
|  | deaminase activity (GO:0019239) | 0.5 |
| *receptor activity (GO:0004872)* | ligand-activated sequence-specific DNA binding RNA polymerase II transcription factor activity (GO:0004879) | 35.7 |
|  | G-protein coupled receptor activity (GO:0004930) | 28.6 |
|  | transmembrane receptor protein tyrosine kinase activity (GO:0004714) | 14.3 |
|  | glutamate receptor activity (GO:0008066) | 14.3 |
|  | GABA receptor activity (GO:0016917) | 7.1 |
| **Biological Process** | | |
| \| *metabolic process (GO:0008152)* \| primary metabolic process (GO:0044238) \| 36.8 \| \| --- \| --- \| --- \| \| nitrogen compound metabolic process (GO:0006807) \| 21.1 \| \| phosphate-containing compound metabolic process (GO:0006796) \| 15.9 \| \| biosynthetic process (GO:0009058) \| 13.3 \| \| catabolic process (GO:0009056) \| 10.9 \| \| coenzyme metabolic process (GO:0006732) \| 0.7 \| \| sulfur compound metabolic process (GO:0006790) \| 0.6 \| \| generation of precursor metabolites and energy (GO:0006091) \| 0.6 \| \| vitamin metabolic process (GO:0006766) \| 0.2 \| \| *biological regulation (GO:0065007)* \| regulation of nucleobase-containing compound metabolic process (GO:0019219) \| 42.6 \| \| regulation of phosphate metabolic process (GO:0019220) \| 33.8 \| \| regulation of cell cycle (GO:0051726) \| 8.8 \| \| negative regulation of apoptotic process (GO:0043066) \| 8.8 \| \| regulation of gene expression, epigenetic (GO:0040029) \| 2.9 \| \| regulation of translation (GO:0006417) \| 2.9 \| \| *response to stimulus (GO:0050896)* \| response to stress (GO:0006950) \| 40 \| \| response to external stimulus (GO:0009605) \| 16.9 \| \| response to endogenous stimulus (GO:0009719) \| 10.8 \| \| immune response (GO:0006955) \| 9.2 \| \| response to abiotic stimulus (GO:0009628) \| 6.2 \| \| cellular defense response (GO:0006968) \| 6.2 \| \| behavior (GO:0007610) \| 6.2 \| \| response to toxic substance (GO:0009636) \| 1.5 \| \| response to biotic stimulus (GO:0009607) \| 1.5 \| \| defense response to bacterium (GO:0042742) \| 1.5 \| | | |
| **Cellular Component** | | |
| *cell part (GO:0044464)* | intracellular (GO:0005622) | 75.3 |
|  | plasma membrane (GO:0005886) | 17.7 |
|  | cell projection (GO:0042995) | 4 |
|  | nuclear outer membrane-endoplasmic reticulum membrane network (GO:0042175) | 2.4 |
|  | basal part of cell (GO:0045178) | 0.3 |
|  | neuronal cell body (GO:0043025) | 0.3 |
| *membrane (GO:0016020)* | plasma membrane (GO:0005886) | 53.7 |
|  | integral to membrane (GO:0016021) | 33.3 |
|  | nuclear outer membrane-endoplasmic reticulum membrane network (GO:0042175) | 7.4 |
|  | postsynaptic membrane (GO:0045211) | 2.8 |
|  | mitochondrial inner membrane (GO:0005743) | 1.9 |
|  | presynaptic membrane (GO:0042734) | 0.9 |
| *synapse (GO:0045202)* | postsynaptic membrane (GO:0045211) | 60 |
|  | neuromuscular junction (GO:0031594) | 20 |
|  | presynaptic membrane (GO:0042734) | 20 |

**Table S3. Top 20 enriched KEGG pathways of the differentially methylated CpGs in AUD subjects**.

| **Term** | **Overlap** | **P-value** | **Adjusted P-value** | **Z-score** | **Genes** |
| --- | --- | --- | --- | --- | --- |
| Calcium signaling pathway_Homo sapiens_hsa04020 | 18/180 | 7.00E-04 | 9.55E-02 | -1.96 | PDGFRB;RYR1;CAMK2B;NOS3;ITPR1;ITPR2;PRKCA;CACNA1D;ATP2A1;TACR1;ADCY7;CACNA1E;MYLK4;ITPKB;CACNA1I;PPP3CA;STIM1;ERBB2 |
| Type II diabetes mellitus_Homo sapiens_hsa04930 | 08/48 | 8.72E-04 | 9.55E-02 | -1.96 | ABCC8;PRKCE;CACNA1D;PIK3R2;PRKCZ;CACNA1E;MTOR;HK2 |
| Adherens junction_Homo sapiens_hsa04520 | 10/74 | 1.13E-03 | 9.55E-02 | -1.80 | TCF7L2;SMAD4;SMAD3;ERBB2;CSNK2A2;PTPRM;PTPRJ;FYN;SORBS1;BAIAP2 |
| Pathways in cancer_Homo sapiens_hsa05200 | 30/397 | 1.81E-03 | 9.55E-02 | -2.03 | PTGER4;LAMA1;LAMA4;PIK3R2;ADCY7;ETS1;RXRB;GNA13;MECOM;MYC;ERBB2;WNT3;RUNX1T1;EGLN1;PDGFRB;TCF7L2;SMAD4;ARHGEF12;SMAD3;BAD;STAT1;PRKCA;MTOR;NFKB2;FGF14;TRAF3;GNB1;RARA;CRK;FGF12 |
| Platelet activation_Homo sapiens_hsa04611 | 13/122 | 2.11E-03 | 9.55E-02 | -1.81 | ARHGEF12;NOS3;ITPR1;ITPR2;PIK3R2;MAPK14;ADCY7;PRKCZ;MYLK4;GNA13;STIM1;FYN;TLN2 |
| Aldosterone synthesis and secretion_Homo sapiens_hsa04925 | 10/78 | 2.27E-03 | 9.55E-02 | -1.87 | CAMK2B;CACNA1I;LIPE;PRKCE;ITPR1;ITPR2;PRKCA;CACNA1D;ADCY7;CREB5 |
| Inflammatory mediator regulation of TRP channels_Homo sapiens_hsa04750 | 11/98 | 3.01E-03 | 9.55E-02 | -1.84 | CAMK2B;PTGER4;PRKCH;PRKCE;ITPR1;ITPR2;PRKCA;PIK3R2;MAPK14;ASIC2;ADCY7 |
| Phosphatidylinositol signaling system_Homo sapiens_hsa04070 | 11/98 | 3.01E-03 | 9.55E-02 | -1.67 | INPP4A;ITPKB;INPP5A;ITPK1;ITPR1;PIP4K2A;ITPR2;PRKCA;PIP5K1B;PIK3R2;DGKH |
| Focal adhesion_Homo sapiens_hsa04510 | 17/202 | 5.95E-03 | 1.51E-01 | -1.72 | PDGFRB;BAD;LAMA1;LAMA4;PRKCA;PIK3R2;THBS1;MYLK4;DIAPH1;ITGA11;ERBB2;RAPGEF1;CAPN2;COL9A3;FYN;TLN2;CRK |
| Proteoglycans in cancer_Homo sapiens_hsa05205 | 17/203 | 6.24E-03 | 1.51E-01 | -1.78 | CAMK2B;ARHGEF12;ITPR1;ITPR2;PRKCA;PIK3R2;ANK3;MAPK14;HSPG2;ESR1;THBS1;MTOR;MYC;ERBB2;SDC1;EIF4B;WNT3 |
| Wnt signaling pathway_Homo sapiens_hsa04310 | 13/142 | 7.72E-03 | 1.51E-01 | -1.63 | CAMK2B;TCF7L2;SMAD4;SMAD3;CSNK2A2;PRKCA;CSNK1E;NKD2;PPP3CA;DAAM1;TBL1XR1;MYC;WNT3 |
| Oxytocin signaling pathway_Homo sapiens_hsa04921 | 14/158 | 7.72E-03 | 1.51E-01 | -1.68 | RYR1;CAMK2B;NOS3;CACNA2D1;ITPR1;ITPR2;PRKAG2;PRKCA;CACNA1D;PIK3R2;PRKAB1;ADCY7;MYLK4;PPP3CA |
| Cholinergic synapse_Homo sapiens_hsa04725 | 11/111 | 7.74E-03 | 1.51E-01 | -1.72 | CAMK2B;GNB1;ITPR1;ITPR2;KCNQ4;PRKCA;CACNA1D;PIK3R2;FYN;ADCY7;CREB5 |
| Acute myeloid leukemia_Homo sapiens_hsa05221 | 07/57 | 1.03E-02 | 1.73E-01 | -1.51 | TCF7L2;BAD;MYC;RARA;PIK3R2;MTOR;RUNX1T1 |
| Regulation of actin cytoskeleton_Homo sapiens_hsa04810 | 17/214 | 1.03E-02 | 1.73E-01 | -1.57 | CYFIP2;PDGFRB;NCKAP1;GSN;ARHGEF12;PIK3R2;BAIAP2;MYLK4;GNA13;DIAPH1;FGF14;ITGAD;ITGA11;PIP4K2A;PIP5K1B;FGF12;CRK |
| AGE-RAGE signaling pathway in diabetic complications_Homo sapiens_hsa04933 | 10/101 | 1.09E-02 | 1.73E-01 | -1.63 | DIAPH1;SMAD4;SMAD3;NOS3;STAT1;PRKCE;PRKCA;PIK3R2;MAPK14;PRKCZ |
| ErbB signaling pathway_Homo sapiens_hsa04012 | 09/87 | 1.17E-02 | 1.75E-01 | -1.42 | CAMK2B;NRG3;BAD;MYC;ERBB2;PIK3R2;PRKCA;CRK;MTOR |
| Vascular smooth muscle contraction_Homo sapiens_hsa04270 | 11/120 | 1.35E-02 | 1.86E-01 | -1.41 | GNA13;PRKCH;ARHGEF12;PRKCE;ITPR1;PLA2G3;ITPR2;PRKCA;CACNA1D;ADCY7;MYLK4 |
| Hippo signaling pathway_Homo sapiens_hsa04390 | 13/153 | 1.39E-02 | 1.86E-01 | -1.30 | TCF7L2;SMAD4;SMAD3;BMP8B;SCRIB;CSNK1E;LIMD1;PRKCZ;SMAD7;FRMD6;DLG2;MYC;WNT3 |
| Insulin signaling pathway_Homo sapiens_hsa04910 | 12/139 | 1.58E-02 | 2.00E-01 | -1.31 | RPTOR;LIPE;BAD;RAPGEF1;PRKAG2;PIK3R2;SORBS1;PRKAB1;PRKCZ;CRK;HK2;MTOR |

**Table S4. IPA^®^ networks**

| **ID** | **Genes** | **Score** | **Focus Molecules** | **Top Diseases and Functions** |
| --- | --- | --- | --- | --- |
| 1 | ARHGEF12,BAD,CACTIN,CaMKII,CCL22,CDK18,Creb,CSNK2A2,DYNLL1,FLII,FUCA1,GRAP2,IFNBeta,IRAK2,ITCH,LITAF,MAD1L1,MDK,MICA,MICU1,NFkB(complex),NUP98,Pka,PTGER4,PTPRS,RIPK4,SIVA1,SMAD7,SULF2,TAB2,TAC1,TACR1,TAGLN,TRAF3,WWOX | 38 | 30 | Cellular Development, Cellular Growth and Proliferation, Hematological System Development and Function |
| **2** | 26sProteasome,ADARB2,CAP2,CAPN2,CORO1C,CTDP1,CTNNBL1,CyclinE,ERCC3,ERCC6,ESR1,FGF12,GTF2H4,KCNK6,LRPPRC,MAEA,MED23,MINDY3,NCOR2,NFKB2,**NR3C1**,POLR1B,PSMB5,RARA,RNA polymerase II,SAP30BP,SEC16A,SEMA5B,SETD2,SHANK2,SP100,TFIIH,TMOD1,TTLL11,Ubiquitin | 38 | 30 | **Drug Metabolism**, Lipid Metabolism, Small Molecule Biochemistry |
| 3 | ABCC4,Alphatubulin,ATAD2,C9orf3,CLU,CP,CYFIP2,CYP27B1,DLEU1,E2f,Hdac,Hsp70,KCNG1,MFGE8,MPP6,MYC,NCKAP1,NDRG1,PDGFBB,PHF21A,RNF220,SMAD3,SMAD4,Smad2/3,TCF7L2,TGM2,THBS1,TINAGL1,TMBIM6,TP63,USP36,WDR5,ZIC2,ZMIZ1,ZNF160 | 36 | 29 | Embryonic Development, Organismal Development, Tissue Development |
| 4 | ACAT1,Actin,ADGRB1,ANTXR1,ARHGEF28,c-Src,CDC16,CDH15,DAAM1,DIAPH1,EGLN,ERBB2,ERK,estrogen receptor,ETS1,GLRX,GNA13,LTBP2,Mek,MMP15,Notch,PCDH7,POLA2,POLE2,PRKCA,PRKCZ,PTPRN2,RHOBTB3,S100A11,SMURF1,SPAG4,SRFBP1,TEP1,TOP1MT,WNT3 | 34 | 28 | Cancer, Gastrointestinal Disease, Hepatic System Disease |
| 5 | ADCY7,ARHGAP22,ARL6IP5,ATP9A,CEP170,Cg,CTNND2,DGKH,FSH,GNB1,Histone h3,Hsp90,Interferon alpha,ITPR1,ITPR2,Jnk,LARP1B,Lh,LIMD1,LMO2,LONP2,NDUFAF6,PLXNC1,POU5F1,PRKCH,RABGAP1L,RUNX1T1,SDF4,SPATS2L,SYNPO,TCF,TRANK1,TTC28,WDR1,ZNF423 | 32 | 27 | Cell Cycle, Cellular Assembly and Organization, Cellular Function and Maintenance |
| 6 | Alpha catenin,Ap1,CALR,CD3,CDH24,DCTN1,DPP4,ERK1/2,FOXP1,GALNT2,GRK5,HK2,HSPG2,HTATIP2,IQSEC1,LDL,MADD,MAP4,MAP2K1/2,Mapk,NCAN,NCOA5,p70 S6k,PACSIN2,PDGFRB,PI3K (complex),PLPP2,PP2A,PTPRJ,Ras,Rock,S1PR2,Shc,STK39,TNS1 | 23 | 22 | Cardiovascular System Development and Function, Cellular Movement, Cardiovascular Disease |
| 7 | 14-3-3,Akt,ASXL1,C1QBP,calpain,caspase,CCDC88A,CORO1B,  DAXX,EIF4B,Gsk3,HDAC10,HIPK2,Histone h4,Hsp27,MAGI2,MAPK14,MTOR,MTORC1,PABPC1,PARP,PIK3R2,PIK3R6,Pkc(s),Ppp2c,PRKAA,PSMB3,Rac,RPTOR,RUNX3,STIM1,TMEM219,TOLLIP,TPD52L1,XRCC5 | 23 | 22 | Cellular Compromise, Cellular Movement, Cell Cycle |
| 8 | ADAM17,BCR (complex),BMPER,C1S,CRK,DAPP1,EGLN1,FFAR4,  HDAC4,Ifngamma,IgG,Igm,IL12complex),Immunoglobulin,MAFF,MAP1LC3,MAP1S,NAIP,P38 MAPK,PI3K(family),PRKCE,Rap1,RAPGEF1,RAPGEF4,RECK,  SDC1,SRC(family),STAT1,TCR,TECPR1,TRIO,Vegf,WARS,ZMPSTE24,ZNF148 | 23 | 22 | Skeletal and Muscular Disorders, Skeletal and Muscular System Development and Function, Cell Morphology |
| 9 | ABL2,ACKR3,ADNP,ARID1A,ARID1B,BABAM2,CCRL2,DUSP13,E2F1,E2F7,EML4,EYA2,EYA4,FER1L6,FFAR2,HLX,ID3,IL15RA,KANK1,MAFF,MARCH1,MFAP1,NME7,NTNG1,PHLDA2,RBMS1,REST,SCG5,SERTAD1,SMARCA4,SOX7,SRRM4,SUZ12,TREM1,UIMC1 | 14 | 16 | Developmental Disorder, Hereditary Disorder, Neurological Disease |
| 10 | ALMS1,ANKRD13A,C1QBP,CACNA1E,CHKA,CTBS,DCDC2,DNAAF4,EFNA1,EGFR,ENAH,ERBB4,EZH2,FHOD3,GORASP1,GRB2,hemoglobin,KIF3A,L3MBTL3,LCN2,MATR3,miR-26a-5p (and other miRNAs w/seed UCAAGUA),NCOA1,NTM,PGR,PGS1,PIP5K1B,RFX1,RFX2,SH3GLB1,SMG6,SSRP1,STRN,TFRC,TMTC1 | 14 | 16 | Cellular Development, Cellular Growth and Proliferation, Renal and Urological System Development and Function |
| 11 | Akt,ANK3,BRAF,BRCA1,CCNA2,CDC25C,CDH1,CDK1,CEP97,CREB5,DLG1,DNMT1,DNMT3B,ERK1/2,ETV6,GABBR1,KBTBD7,KIF13B,LHFPL2,Mek,MYC,NEK6,NEK9,NGFR,NUPR1,PRKACA,RAB39B,RFTN2,RLN3,SAFB,SPDL1,TP53,XBP1,ZNF217,ZNF488 | 12 | 15 | Cell Cycle, Cancer, Organismal Injury and Abnormalities |
| 12 | ADGRG3,BAP1,CCNE2,CITED2,CYP17A1,DENND1A,DNAJC6,FDFT1,FOXK1,GHR,GNA13,HEXIM1,INPP4A,MDC1,MLF2,MPZL2,MYL9,NAV1,NFIC,P-TEFb,PDLIM2,PLXNB2,PPP1R13L,PPP3CA,PRKAB1,RHOA,SMURF1,STARD13,STMN1,TP53,TRIM24,TRIM28,WWOX,YPEL3,ZNF300 | 12 | 15 | Organismal Survival, Cell Death and Survival, Cancer |
| 13 | ABLIM1,ACRBP,CD44,CLDN7,CST5,CTSB,DCTN1,DTNB,EPCAM,EPHA2,EZR,F3,GABRA1,GANAB,GC,GTF2H3,IFI44,INTS1,INTS4,INTS10,ITPR3,LIMD2,NUDT21,NUMA1,PCBP1,PIP4K2A,POLR2B,PTBP1,RRP1,SLC43A2,SLC7A11,SYVN1,TOMM40,VSNL1,XXYLT1 | 12 | 15 | RNA Post-Transcriptional Modification, Cell Cycle, Cancer |
| 14 | ANKRD17,ARHGAP10,ARSB,BAIAP2,CBS/CBSL,CCNA2,CPSF6,DCPS,EIF3M,EXOSC4,HAS2-AS1,HDAC1,HDAC4,HSD11B2,IL7R,IRF4,ITGAD,KRT81,LHCGR,MANF,MAOB,MAT2B,MIER1,NCF2,OGG1,PADI4,PEX14,PLEKHA5,PREX1,SLC39A8,SP1,SP3,WNT9A,XYLT1,YWHAG | 12 | 15 | Free Radical Scavenging, Cell Morphology, Hematological System Development and Function |
| 15 | ACADS,ADH5,BUB1,CBS/CBSL,CCNB1,CPT1A,CYB5R3,glutathione peroxidase,GOLGA3,Growth hormone,INS,ITGA11,LONP1,LRPPRC,mir-29,miR-1-3p (and other miRNAs w/seed GGAAUGU),MRPS31,NDC80,NEK2,POLD1,POLE2,PRC1,PRIM1,PRKAG2,PTPRM,RAB1B,RABL6,RFC3,SEC24D,SLC30A8,TMEM97,TP53,TRAP1,TRIP13,ZYG11A | 12 | 15 | Gastrointestinal Disease, Hepatic System Disease, Liver Steatosis |
| 16 | AKR1B1,APOC2,APOE,BTK,COL16A1,CTSS,ELF3,GPD2,HAS1,HLA-DQ,IFNL2,IL1B,IL4I1,ILK,ITGAX,LAMA1,LBP,LILRA5,  LIMS2,MAPK13,MMP8,NPAS3,PLA2G3,RYR1,SH3BP5,SLC4A1,Sod,SORBS1,TAB2,TAB3,TAC1,TCIM,TNF,WRB,ZNF267 | 11 | 14 | Cellular Development, Hematological System Development and Function, Lymphoid Tissue Structure and Development |
| 17 | AHI1,ARAF,ARSA,CCND1,CDC6,CDC37,CDK6,CLPB,ELAVL1,ERI1,FBN1,FBXO7,FNBP1,FOXP1,HDAC7,JCAD,MAML2,MAML3,MARCKS,MAT2B,MELK,MFSD6,NCAPG,OAS1,OSBPL2,PALMD,PRKCD,PSMD10,PTPRU,RBFOX3,RFC5,RGS2,SERPINB2,SYK,TAL1 | 11 | 14 | Cancer, Organ Morphology, Cellular Development |
| 18 | ACTA1,ADAMTSL5,AGTR1,APOLD1,ARHGEF11,CACNA1C,CACNA1D,CACNA2D1,CACNB3,CLK3,CTTN,ENO1,FURIN,HNRNPK,IL1,IL17A,ISLR2,LCN1,LMCD1,mir-143,mir145,MMP9,MYH11,MYOCD,PALM2-AKAP2,PCSK5,PIAS3,PRDM5,SPIN1,SRF,TAGLN,TNFSF12,TTN,VEGFD,ZFPM1 | 11 | 14 | Cardiovascular Disease, Organismal Injury and Abnormalities, Cardiac Dysfunction |
| 19 | BAG6,CBX4,CBX8,CCNY,CDKN1A,CELF1,CSF1R,CXXC1,ELOA,FOXG1,JARID2,MAPK13,MECOM,NASP,NFAT5,PARG,PARP1,PCGF3,PHC1,PHC2,PHC3,PRDM16,RECQL5,RING1,RNF2,RNF19A,RYBP,SETD1B,TRIM29,UBE3A,UBL4A,VCP,WDR82,XRCC1,YAF2 | 10 | 13 | DNA Replication, Recombination, and Repair, Cell Morphology, Cellular Function and Maintenance |
| 20 | BMP2K,BMP8B,CD2,CD9,CD81,CRKL,DOCK5,ENPEP,EPO,FES,HAMP,HECW1,HNF1A,IL22,INPP5A,INPP5D,JAK1,KALRN,KIT,MAF,MAP3K9,MC2R,MRAP,NFATC2,NPY5R,OLR1,OSM,PTPN11,PTPN18,RASAL1,RNF39,STAT5A,STAT5a/b,THPO,TYK2 | 10 | 13 | Hematological System Development and Function, Immunological Disease, Inflammatory Disease |
| 21 | ACSS2,AHNAK2,AR,CAV1,CAV2,CAVIN1,ELF1,FES,FGFR4,FSCN1,GPS2,HP,ITPKB,LIPE,LRIG1,MED14,MED24,MGEA5,NPC1,Nr1h,NR6A1,PC,PDE9A,PIAS3,PTPN2,PTPRT,SPEN,STAT3,TBL1X,TBL1XR1,TLR4,TPD52L1,TRPC3,VIRMA,ZDHHC17 | 10 | 13 | Cancer, Organismal Injury and Abnormalities, Reproductive System Disease |
| 22 | ASPM,CCDC50,CD24,CMIP,CYLD,DNAJC13,FAT4,FBXW7,GAL3ST1,GAS7,GLI1,GRK2,HIF1A,HUWE1,IFNG,ISG20,MAN1C1,MECR,MIXL1,NCS1,NF1,OAS3,OASL,OGG1,POR,RAB11FIP4,RARRES1,REPIN1,RIPK1,RNASEL,SDC4,SPP1,STOML1,TMEM45A,ULBP2 | 10 | 13 | Cancer, Organismal Injury and Abnormalities, Tissue Morphology |
| 23 | AGO1,AGO2,APBB1,APP,BIN1,CAMTA1,DICER1,GADD45GIP1,GLUL,HMOX1,HSF1,ID3,KAT5,MICA,mir-9,mir-19,mir-199,mir-335,miR-130a-3p (and other miRNAs w/seed AGUGCAA),miR-16-5p (and other miRNAs w/seed AGCAGCA),miR-17-5p (and other miRNAs w/seed AAAGUGC),miR-200b-3p (and other miRNAs w/seed AAUACUG),miR-9-3p (and other miRNAs w/seed UAAAGCU),NANOS1,PCDH15,PDZD2,PICALM,RAD51B,RHBDD1,RIN3,RTN1,SGMS1,SLC40A1,SRA1,TARBP2 | 9 | 12 | Cancer, Organismal Injury and Abnormalities, Reproductive System Disease |
| 24 | ABCC8,ACTN1,ANK2,BCL2,BIRC5,caspase,CDX2,CLDN18,CSF2,DNAJC11,DSTYK,EP300,FAM110A,IL5,IRF2,KCNJ11,LAG3,MEP1A,miR-122-5p (miRNAs w/seedGGAGUGU),NR0B2,NR5A2,NSMCE1,NSMCE2,NSMCE3,  NSMCE4A,PIAS1,RHOA,RIPOR2,SATB1,SLC51A,SMC5,SMC6,SUMO1,UBE2I,VPS37B | 9 | 12 | Cellular Development, Cellular Growth and Proliferation, Cell Death and Survival |
| 25 | ADAP1,AMPK,CD3group,CFAP36,CLUAP1,CRYL1,FOXN3,FYN,HSPB11,IFT20,IFT22,IFT27,IFT46,IFT52,IFT57,IFT74,IFT80,IFT81,IFT88,IFT172,LIME1,LPCAT3,MAPK1,NDUFA10,NKD2,NT5C2,OASL,PACS1,PTK2B,SKAP1,TCR,TGFA,TRAF3IP1,TTC26,TTC30B | 8 | 11 | Cell Signaling, Cell Morphology, Cellular Assembly and Organization |

**Table S5. Genes of the NR3C1 hierarchical interaction network.** ^a^ Chromosome, ^b^ Locations of CpGs within 1,500bp from the TSS (TSS1500), within 200bp from the TSS (TSS200), 5’ untranslated regions (5’ UTRs), gene bodies (Body), first exon (1stExon). ^c^ Log-scale fold changes obtained from the R *limma* package (v 3.32.5). ^d^ Nominal *p*-value obtained from the *limma* package. ^e^ FDR values obtained by multiple testing. Genes highlighted in bold have been validated by (h)MeDIP assay.

| Gene  Symbol | CpG | Chr^a^ | Position | Location^b^ | logFC^c^ | P_nominal_^d^ | FDR^e^ |
| --- | --- | --- | --- | --- | --- | --- | --- |
| SP100 | cg05876232 | chr2 | 231279916 | TSS1500 | -0.226 | 2.30E-05 | 0.478 |
| SETD2 | cg27106049 | chr3 | 47201188 | Body | 0.137 | 2.74E-05 | 0.509 |
| ERCC3 | cg08491733 | chr2 | 128051434 | Body | 0.231 | 1.27E-04 | 0.618 |
| ERCC6 | cg22442391 | chr10 | 50696342 | Body | 0.244 | 1.38E-04 | 0.646 |
| POLR1B | cg03495769 | chr2 | 113299861 | 5'UTR;1stExon;Body | 0.470 | 1.38E-04 | 0.646 |
| CAPN2 | cg00792251 | chr1 | 223888905 | TSS1500 | -0.179 | 1.84E-04 | 0.655 |
| TTLL11 | cg06708747 | chr9 | 124626545 | Body | 0.170 | 2.02E-04 | 0.655 |
| ESR1 | cg20583095 | chr6 | 152127920 | 5'UTR;TSS1500 | -0.273 | 2.64E-04 | 0.655 |
| SEC16A | cg21233622 | chr9 | 139345761 | Body | 0.169 | 2.77E-04 | 0.655 |
| CTDP1 | cg02044695 | chr18 | 77449125 | 5'UTR;Body | -0.134 | 2.80E-04 | 0.655 |
| NCOR2 | cg19184362 | chr12 | 125034431 | 5'UTR | 0.189 | 3.76E-04 | 0.663 |
| NFKB2 | cg26894311 | chr10 | 104153930 | TSS1500 | 0.162 | 3.85E-04 | 0.663 |
| DNMT3B | cg18730317 | chr20 | 31374593 | Body | 0.186 | 4.03E-04 | 0.663 |
| MAEA | cg08980067 | chr4 | 1289366 | Body | -0.266 | 4.88E-04 | 0.675 |
| PSMB5 | cg20988373 | chr14 | 23502987 | Body;5'UTR | -0.098 | 5.01E-04 | 0.675 |
| LRPPRC | cg26445008 | chr2 | 44150174 | Body | -0.367 | 5.53E-04 | 0.675 |
| GTF2H4 | cg15564965 | chr6 | 30875659 | TSS1500 | -0.203 | 5.60E-04 | 0.675 |
| SHANK2 | cg06601450 | chr11 | 70323814 | Body | 0.142 | 5.78E-04 | 0.675 |
| ADARB2 | cg10542123 | chr10 | 1686470 | Body | 0.181 | 5.93E-04 | 0.678 |
| TMOD1 | cg07755265 | chr9 | 100298376 | Body | -0.400 | 8.07E-04 | 0.695 |
| KCNK6 | cg00259945 | chr19 | 38809881 | TSS1500 | -0.147 | 8.16E-04 | 0.695 |
| CTNNBL1 | cg04903820 | chr20 | 36372888 | Body | 0.208 | 1.00E-03 | 0.703 |
| CAP2 | cg02048478 | chr6 | 17432613 | Body | -0.134 | 1.01E-03 | 0.703 |
| SEMA5B | cg07881714 | chr3 | 122658205 | Body | 0.260 | 1.01E-03 | 0.703 |
| FGF12 | cg17448661 | chr3 | 192431079 | Body | 0.146 | 1.02E-03 | 0.703 |
| CORO1C | cg13204290 | chr12 | 109125409 | TSS200 | 0.387 | 1.06E-03 | 0.703 |
| NR3C1 | cg06521673 | chr5 | 142782072 | 5'UTR | 0.179 | 1.07E-03 | 0.703 |
| RARA | cg13296436 | chr17 | 38474488 | 5'UTR;TSS200 | 0.183 | 1.07E-03 | 0.703 |
| MED23 | cg00405086 | chr6 | 131949743 | TSS1500 | 0.232 | 1.09E-03 | 0.703 |
| SAP30BP | cg07389228 | chr17 | 73685651 | Body | 0.375 | 1.09E-03 | 0.703 |
| SHANK2 | cg05337228 | chr11 | 70419071 | Body | 0.144 | 1.63E-03 | 0.715 |
| SHANK2 | cg27066203 | chr11 | 70451047 | Body | 0.366 | 1.65E-03 | 0.715 |
| NCOR2 | cg01400924 | chr12 | 124947742 | Body | 0.149 | 1.76E-03 | 0.715 |
| CORO1C | cg16646466 | chr12 | 109125397 | TSS200 | -0.329 | 1.78E-03 | 0.715 |
| NCOR2 | cg15368151 | chr12 | 124849727 | Body | 0.164 | 1.92E-03 | 0.721 |
| CAPN2 | cg02817106 | chr1 | 223915777 | Body | -0.188 | 1.95E-03 | 0.721 |
| ADARB2 | cg10591346 | chr10 | 1356936 | Body | 0.136 | 1.99E-03 | 0.721 |
| NCOR2 | cg07954091 | chr12 | 124864913 | Body | 0.167 | 2.12E-03 | 0.721 |
| NFKB2 | cg09269103 | chr10 | 104155497 | 5'UTR;1stExon | -0.296 | 2.43E-03 | 0.729 |
| NCOR2 | cg14031846 | chr12 | 124896614 | Body | 0.217 | 2.54E-03 | 0.729 |
| SHANK2 | cg23613253 | chr11 | 70440347 | Body | 0.161 | 3.24E-03 | 0.747 |
| SETD2 | cg19960185 | chr3 | 47124414 | Body | 0.150 | 3.26E-03 | 0.747 |
| SHANK2 | cg11999601 | chr11 | 70670999 | Body | 0.111 | 3.55E-03 | 0.747 |
| ADARB2 | cg08511578 | chr10 | 1403938 | Body | 0.148 | 3.59E-03 | 0.747 |
| CORO1C | cg23749353 | chr12 | 109083899 | Body | 0.096 | 4.19E-03 | 0.748 |
| ESR1 | cg19449067 | chr6 | 152011103 | TSS1500 | 0.229 | 4.24E-03 | 0.748 |
| ADARB2 | cg16772023 | chr10 | 1308866 | Body | 0.166 | 4.32E-03 | 0.748 |
| SHANK2 | cg13756965 | chr11 | 70917458 | 5'UTR | 0.156 | 4.39E-03 | 0.748 |
| SHANK2 | cg08033640 | chr11 | 70419197 | Body | -0.212 | 4.76E-03 | 0.748 |

**Table S6. Validation of the NR3C1 hierarchical interaction network genes by methylated (MeDIP) and hydroxymethylated DNA Immunoprecipitation (hMeDIP) assays.** Data are expressed as fold change of controls mean ± SEM. * *p* <0.05, Student’s t-test *vs.* controls.

| **Gene Symbol** | **MedIP** | | **hMedIP** | |
| --- | --- | --- | --- | --- |
|  | **Control** | **AUD** | **Control** | **AUD** |
| SP100 | 1.00±0.06 | 1.06±0.08 | 1.00±0.10 | 0.76±0.05* |
| POLR1B | 1.00±0.10 | 1.35±0.14* | 1.00±0.08 | 0.77±0.11 |
| LRPPRC | 1.00±0.12 | 0.97±0.09 | 1.00±0.07 | 0.76±0.05* |
| TMOD1 | 1.00±0.06 | 0.93±0.06 | 1.00±0.09 | 0.72±0.06* |
| SEMA5B | 1.00±0.09 | 1.26±0.08* | 1.00±0.08 | 0.78±0.05* |
| NR3C1 | 1.00±0.12 | 0.89±0.17 | 1.00±0.09 | 1.31±0.09* |
| SAP30BP | 1.00±0.09 | 1.31±0.11* | 1.00±0.12 | 0.66±0.05* |

**Table S7: MECP2 and DNMT1 binding status to NR3C1 exon 1 in the prefrontal cortex (BA10) of alcohol use disorder (AUD) subjects assessed by chromatin immunoprecipitation assay (ChIP).** Values are expressed as mean ± SEM of fold change of control of 25 controls and 24 AUD subjects for ChIP. MECP2 binding (1_B_ -4068 to -3939: t_1,47_ = 2.64, *p* = 0.011; 1_H_ -1701 to -1553: t_1,47_ = 2.34, *p* = 0.022; 1_H_ -2382 to -2308: t_1,47_ = 2.30, *p* = 0.026; 1_H_ -2071 to -1952: t_1,47_ = 3.01, *p* = 0.004). DNMT binding (1_B_ -4068 to -3939: t_1,47_ = 1.53, *p* = 0.133; 1_H_ -2382 to -2308: t_1,47_ = 2.51, *p* = 0.016; 1_H_ -2071 to -1952: t_1,47_ = 2.46, *p* = 0.018). * *p*<0.05, ** *p*<0.01, Student’s t-test *vs*. controls.

| **NR3C1 exon 1** | **DNMT1 Binding** | **MECP2 Binding** |
| --- | --- | --- |
| 1_B_ -4068 to -3939 | 0.74 ± 0.08 | 0.59 ± 0.08* |
| 1_H_ -1701 to - 1553 | N/A | 0.63 ± 0.11* |
| 1_H_ -2382 to -2302 | 0.65 ± 0.09* | 0.65 ± 0.10* |
| 1_H_ -2071 to -1952 | 0.61 ± 0.07* | 0.56 ± 0.06** |


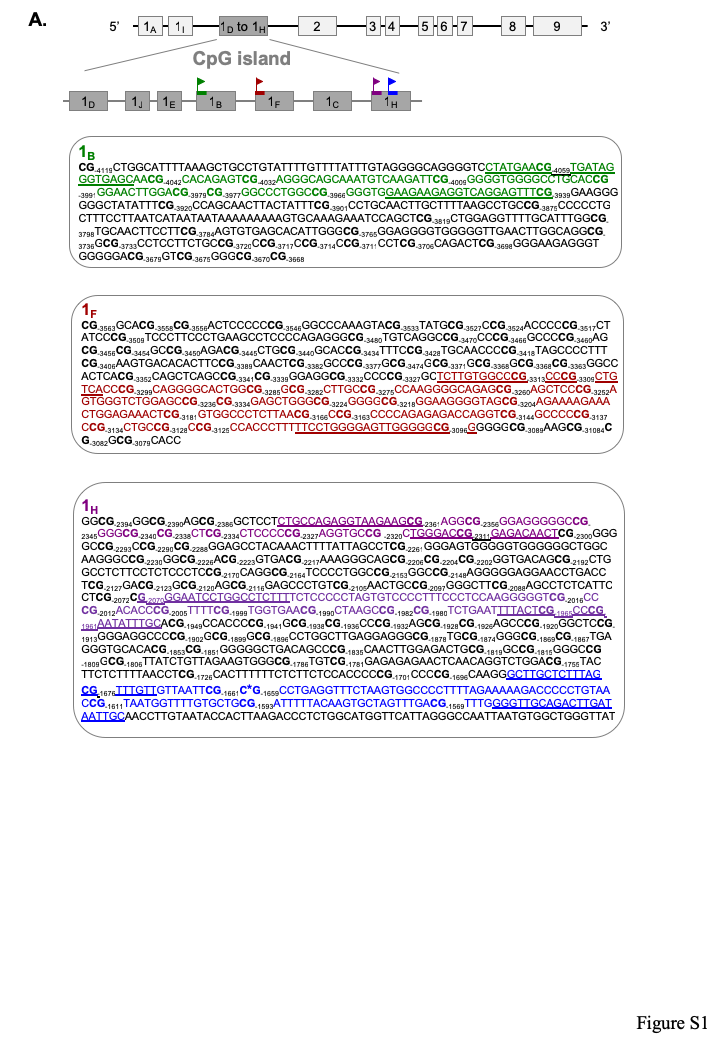


**Figure S1. Human *NR3C1* gene structure.** Each rectangle is an exon, the CpG island is illustrated as dark grey rectangles. Flag signs indicate the regions of the gene amplified after (hydroxy)methylated DNA immunoprecipitation [(h)MeDIP] assay or chromatin immunoprecipitation (ChIP) in the untranslated alternative first exons. Sequence of the exon 1_B_, 1_F_ and 1_H_ are presented in the lower panel. Underlined sequences represent primers used for PCR amplification. * indicates the position of the significant differentially methylated CpG observed in the microarray.

**
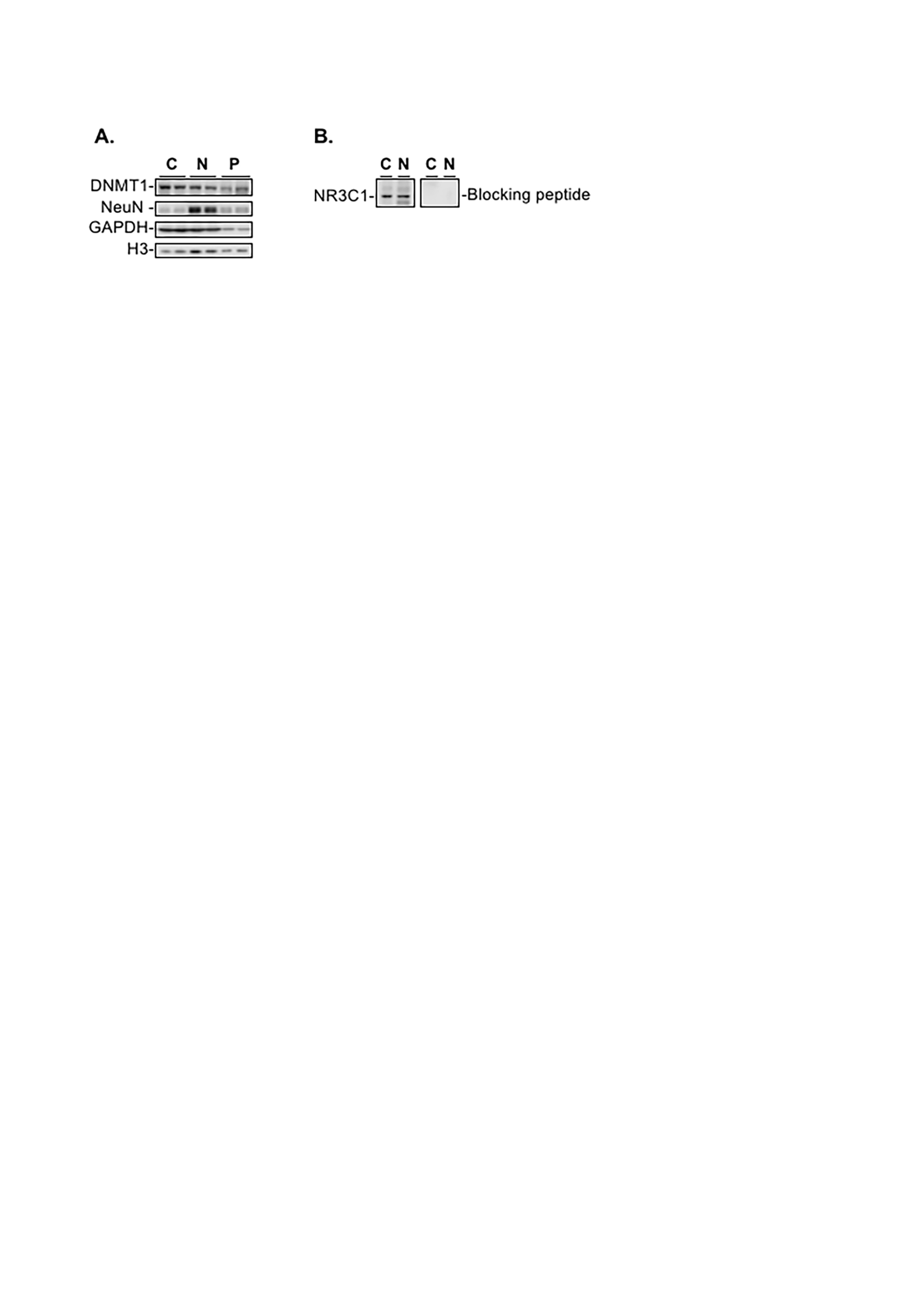
**

**Figure S2. Western Blot Validation.** (C) cytosolic fraction, (N) soluble nuclear fraction, (P) insoluble nuclear pellet. (A) Subcellular fraction validation. DNA Methyltransferase 1 (DNMT1), Neuronal Nuclei Antigen (NeuN), Glyceraldehyde-3-Phosphate Dehydrogenase (GAPDH), H3 Histone Family Member (H3). (B) Anti-glucocorticoid receptor (NR3C1) blocking peptide.

**
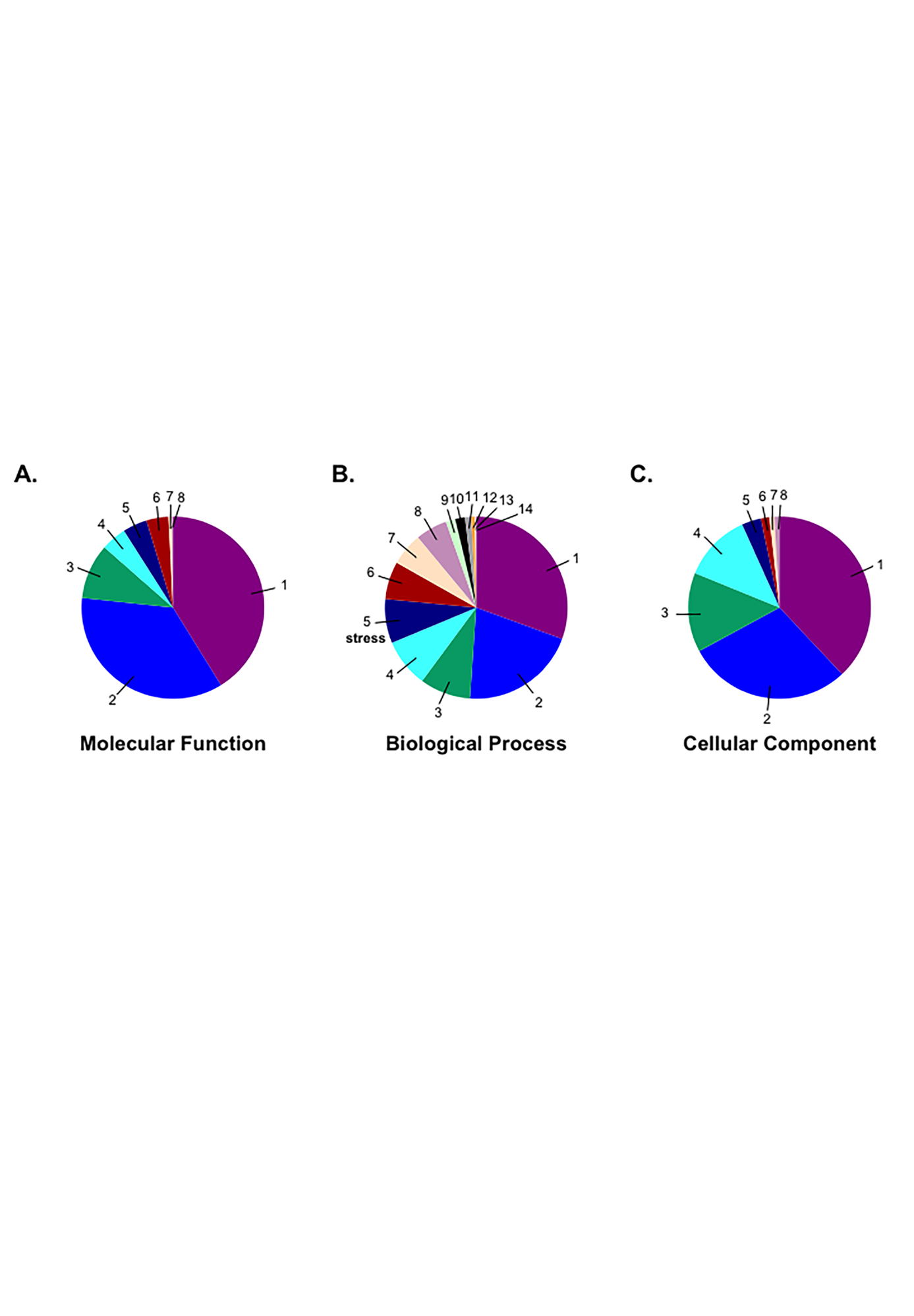
**

**Figure S3. Gene Ontology analysis.** (A) molecular function (1. binding (GO:0005488) 41.2%, 2. catalytic activity (GO:0003824) 35.5%, 3. transporter activity (GO:0005215) 9.8%, 4. receptor activity (GO:0004872) 4.5%, 5. signal transducer activity (GO:0004871) 4.3%, 6. structural molecule activity (GO:0005198) 3.9%, 7. translation regulator activity (GO:0045182) 0.6%, 8. channel regulator activity (GO:0016247) 0.2%); (E) biological process (1. cellular process (GO:0009987) 30.6%, 2. metabolic process (GO:0008152) 20.5 %, 3. biological regulation (GO:0065007) 9%, 4. cellular component organization or biogenesis (GO:0071840) 8.6%, 5. response to stimulus (GO:0050896) 7.8%, 6. localization (GO:0051179) 6.7%, 7. multicellular organismal process (GO:0032501) 5.8%, 8. developmental process (GO:0032502) 5.6%, 9. biological adhesion (GO:0022610) 1.7%, 10. immune system process (GO:0002376) 1.7%, 11. locomotion (GO:0040011) 1.1%, 12. reproduction (GO:0000003) 0.7%, 13. growth (GO:0040007) 0.1%, 14. cell killing (GO:0001906) 0.1%) and (F) Cellular component (1. cell part (GO:0044464) 38%, 2. organelle (GO:0043226) 29%, 3. macromolecular complex (GO:0032991) 14.1%, 4. membrane (GO:0016020) 12.1%, 5. extracellular region (GO:0005576) 3.4%, 6. cell junction (GO:0030054) 1.5%, 7. synapse (GO:0045202) 0.9%, 8. extracellular matrix (GO:0031012) 0.9%).

**
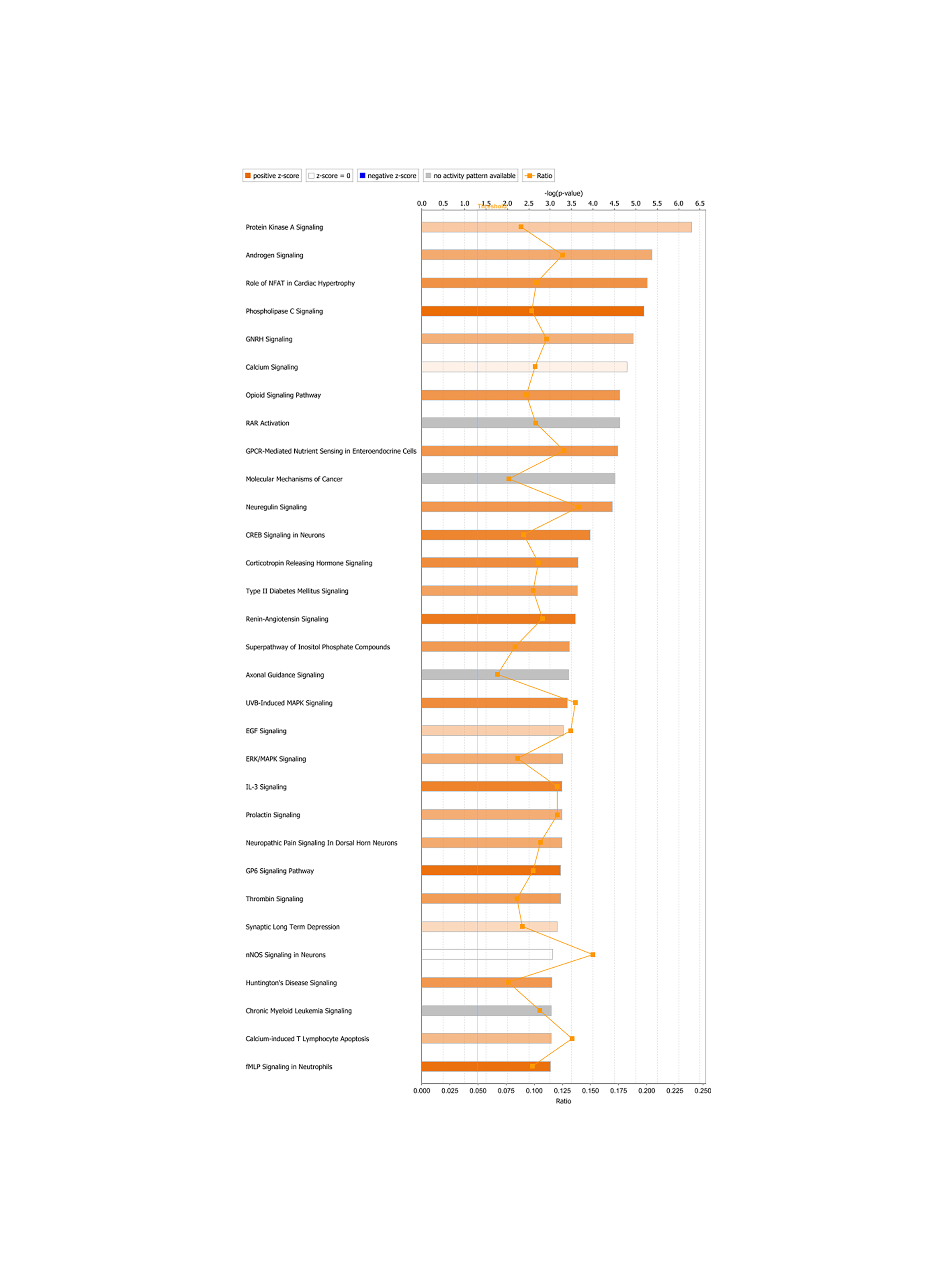
**

**Figure S4. Canonical IPA^®^ pathways of differentially methylated CpGs in AUD subjects.**

The significant Canonical Pathways enriched from our data-set are displayed along the x-axis. The y-axis displays the -log of *p*-value which is calculated by Fisher's exact test right-tailed, with taller bars corresponding to increased significance. The bars show predicted pathway activation (orange), or predicted inhibition (blue), respectively (z-score). White bars have a z-score at or very close to 0. Gray bars indicate pathways where no prediction can currently be made.

The line connecting orange points represents ‘Ratio’, which is calculated as follows: number of genes in a given pathway that meet the cutoff criteria [-log (*p*-value) of 1.3], divided by the total number of genes that make up that pathway and that are in the reference gene set.

**
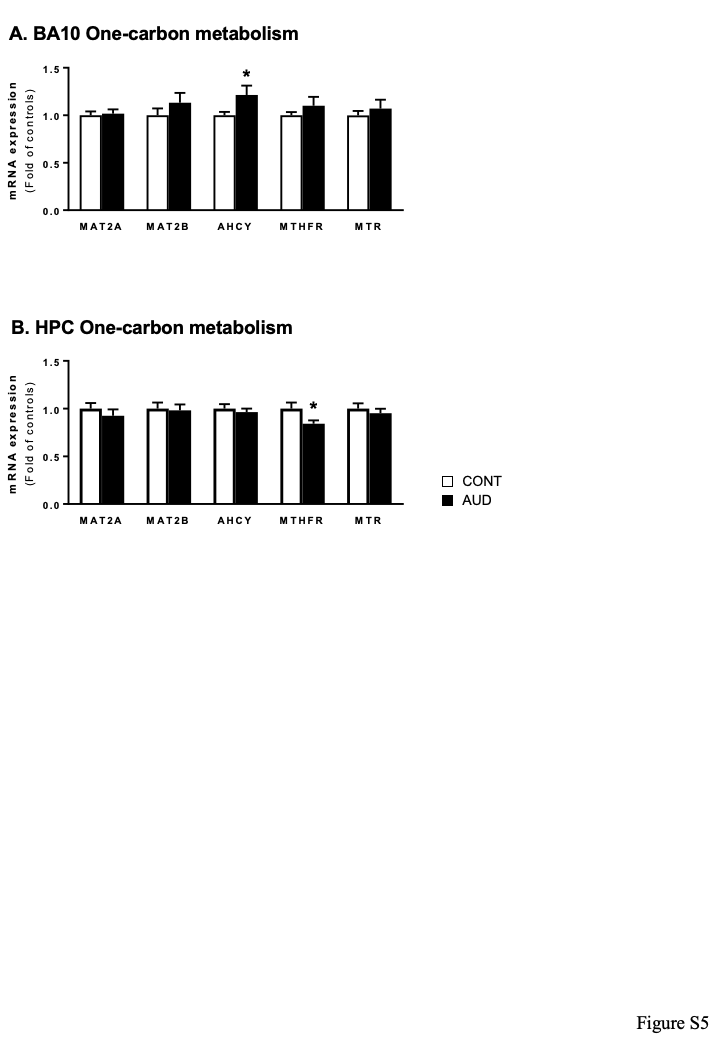
**

**Figure S5: One-carbon metabolism in the prefrontal cortex (BA10) and the hippocampus of alcohol use disorder (AUD) subjects.**

mRNA levels of methionine adenosyltransferase (MAT) 2A, MAT2B, adenosyl homocysteine hydrolase (AHCY), methylenetetrahydrofolate reductase (MTHFR) and methionine synthase (MTR) in (A) BA10 (AHCY: t_1,46_ = 2.07, *p* = 0.044) and (B) hippocampus (MTHFR: t_1,46_ = 2.20, *p* = 0.033). Values are mean ± SEM of 24 samples per group. * p<0.05, Student’s t-test *vs*. controls.

**
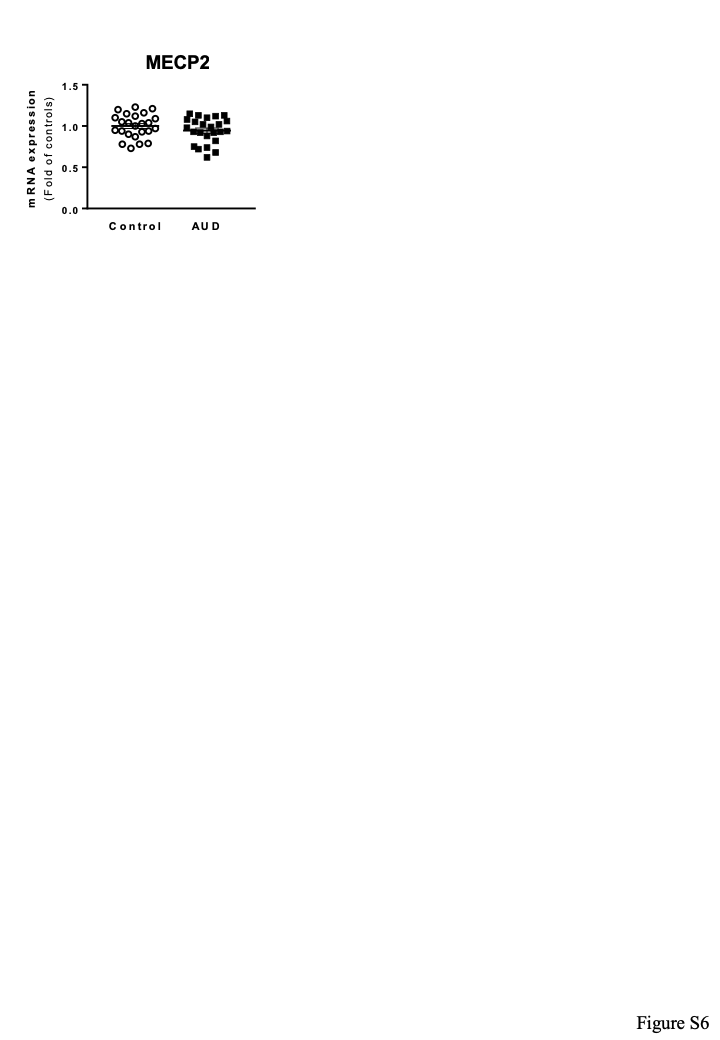
**

**Figure S6: Methyl-CpG binding protein 2 (MECP2) mRNA levels**

Values are mean ± SEM of 24 samples per group.

**
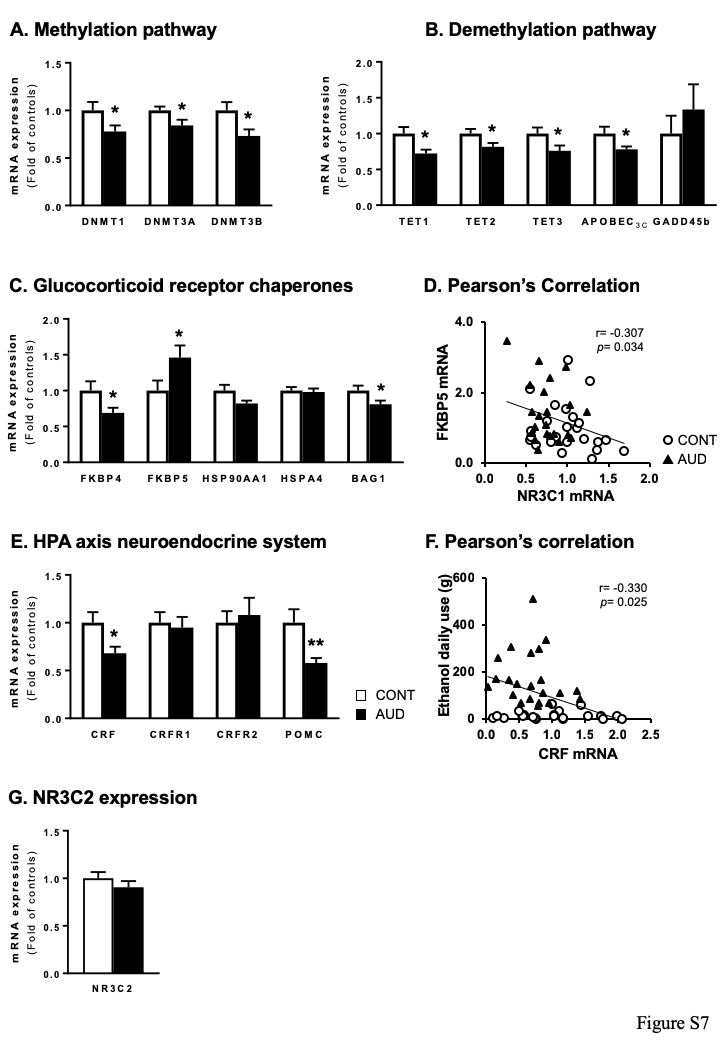
**

**Figure S7: Transcriptomic changes in the stress system and DNA transmethylation reactions in the hippocampus of alcohol use disorder (AUD) subjects.**

mRNA levels of (A) enzymes of the DNA methylation pathway, i.e. DNA methyltransferases (DNMT) 1 (t_1,46_ = 2.02, *p* = 0.049), 3A (t_1,46_ = 2.19, *p* = 0.034) and 3B (t_1,46_ = 2.43, *p* = 0.019). These data remained significant after controlling for FDR (adjusted *p*-value = 0.054)*.* (B) Enzymes involved in DNA demethylation pathway, such as Ten-Eleven Translocase (TET) 1 (t_1,46_ = 2.62, *p* = 0.012), -2 (t_1,46_ = 2.19, *p* = 0.034) and -3 (t_1,46_ = 2.13, *p* = 0.039), as well as Apolipoprotein B mRNA editing enzyme catalytic subunit 3C (APOBEC 3C, t_1,46_ = 2.14, *p* = 0.038) and Growth Arrest and DNA Damage Inducible Beta (GADD45B). These data remained significant after controlling for FDR (adjusted *p*-value = 0.011). (C) Glucocorticoid receptor cytosolic chaperones: FK506 Binding Protein 4 (FKBP4, t_1,46_ = 2.13, *p* = 0.038) and 5 (FKBP5, t_1,46_ = 2.404, *p* = 0.0147), Heat Shock Protein 90 Alpha Family Class A Member 1(HSP90AA1), Heat Shock Protein Family A (Hsp70) Member 4 (HSPA4), BCL2 Associated Athanogene 1 (BAG1, t_1,46_ = 2.13, *p* = 0.038). These data remained significant after controlling for FDR (adjusted *p*-value = 0.017). (D) Pearson’s correlation analysis of FKBP5 and NR3C1 mRNA levels. (E) Hypothalamic-pituitary-adrenal (HPA) axis neuroendocrine system: Corticotropin Releasing Factor (CRF, t_1,46_ = 2.36, *p* = 0.023), Corticotropin Releasing Factor Receptor 1 (CRFR1), Corticotropin Releasing Factor Receptor 2 (CRFR2), Proopiomelanocortin (POMC, t_1,46_ = 2.75, *p* = 0.008). These data remained significant after controlling for FDR (CRF adjusted *p*-value = 0.025, POMC adjusted *p*-value = 0.018). (F) Pearson’s correlation analysis of CRF mRNA levels and ethanol daily use (g). (G) Mineralocorticoid receptor (NR3C2) mRNA levels (t_1,46_ = 1.02, *p* = 0.315). Values are mean ± SEM of 24 samples per group. * p<0.05, ** *p*<0.01 Student’s t-test *vs*. controls.
